# Lactulose Improves Neurological Outcomes by Repressing Harmful Bacteria and Regulating Inflammatory Reactions in Mice After Stroke

**DOI:** 10.3389/fcimb.2021.644448

**Published:** 2021-07-13

**Authors:** Quan Yuan, Ling Xin, Song Han, Yue Su, Ruixia Wu, Xiaoxuan Liu, Jimusi Wuri, Ran Li, Tao Yan

**Affiliations:** Department of Neurology, Tianjin Medical University General Hospital, Tianjin Neurological Institute, Key Laboratory of Post-Neurotrauma, Neurorepair, and Regeneration in Central Nervous System, Ministry of Education and Tianjin City, Tianjin, China

**Keywords:** stroke, gut microbiota, lactulose, dysbiosis, microbiota-gut-brain-axis

## Abstract

**Background and Objective:**

Gut microbiota dysbiosis following stroke affects the recovery of neurological function. Administration of prebiotics to counteract post-stroke dysbiosis may be a potential therapeutic strategy to improve neurological function. We aim to observe the effect of lactulose on neurological function outcomes, gut microbiota composition, and plasma metabolites in mice after stroke.

**Methods:**

Male C57BL/6 mice (20–25 g) were randomly divided into three groups: healthy control, photothrombotic stroke + triple-distilled water, and photothrombotic stroke + lactulose. After 14 consecutive days of lactulose administration, feces, plasma, and organs were collected. 16S rDNA sequencing, plasma untargeted metabolomics, qPCR, flow cytometry and Elisa were performed.

**Results:**

Lactulose supplementation significantly improved the functional outcome of stroke, downregulated inflammatory reaction, and increased anti-inflammatory factors in both the brain and gut. In addition, lactulose supplementation repaired intestinal barrier injury, improved gut microbiota dysbiosis, and partially amended metabolic disorder after stroke.

**Conclusion:**

Lactulose promotes functional outcomes after stroke in mice, which may be attributable to repressing harmful bacteria, and metabolic disorder, repairing gut barrier disruption, and reducing inflammatory reactions after stroke.

## Introduction

As technology and science advance, a complex interaction among the brain, gut, and microbiota residing in the gut, which comprises the concept of a microbiota-gut-brain axis, has been gradually accepted (Cryan et al., 2019). Five pathways (Wang and Wang, 2016) related to neuroanatomy, neuroendocrine function, gut immune responses, metabolism, and barriers communicate in the microbiota-gut-brain axis. Stroke causes gut dysfunction, which involves increased intestinal permeability and dysmotility, leading to gut dysbiosis. Increased intestinal permeability might lead to bacterial translocation, which may result in post-stroke complications, such as pneumonia (Stanley et al., 2016). However, the results of dysbiosis after stroke differ in various studies. For example, altered short-chain fatty acid (SCFA)-producing bacteria were observed in several studies. Chuhong Tan et al. (2020) found decreased SCFA-producing bacteria and fecal SCFA levels in acute ischemic stroke patients, while Na Li et al. (2019) found enriched SCFA-producing genera, including *Odoribacter* and *Akkermansia*. Targeting the microbiota-gut-brain axis provides important new directions to treat or prevent stroke and its complications. In fact, therapies involving antibiotics (Benakis et al., 2016; Winek et al., 2016), fecal microbiota transplantation (Spychala et al., 2018; Chen et al., 2019), and prebiotic intervention (Anderson et al., 2009) have been used to treat stroke.

Lactulose, a common prebiotic composed of fructose and galactose, has many potential applications in the food and pharmaceutical industries (Nooshkam et al., 2018). It promotes probiotic bacteria growth, suppresses pathogenic bacteria, and has been widely used to treat hepatic encephalopathy and chronic constipation due to its ability to decrease blood ammonia levels, acidify gut contents, soften stool, and promote bowel movement (Schumann, 2002). Recently, an *in vitro* study explored the prebiotic effect of lactulose under various dosages, and a dose-dependent effect of lactulose on gut microbiota and SCFAs was found (Bothe et al., 2017). Clinically, lactulose is often used to treat post-stroke constipation, with a concentration of 66.7%. Several previous studies (Zhai et al., 2013; Mao et al., 2016; Zhai et al., 2018; Zhang et al., 2019a) showed that a lower concentration of lactulose had beneficial effects on normal or diseased mice. Zheng Zhang et al. (2019a) found that *Bifidobacterium* and *Bacteroides* and many metabolites including SCFAs were significantly increased in pregnant mice after 2 weeks of 15% lactulose intervention. Another study (Zhang et al., 2019b) found that 4 weeks of 15% lactulose intervention significantly lowered blood pressure increased by high-salt diets, decreased inflammatory factor expression, increased the abundance of *Bifidobacterium* and *Alloprevotella*, and altered fecal metabolic profiles. Furthermore, Shixiang Zhai et al.( 2018) found that 3 weeks of lactulose intervention promoted hydrogen-producing bacteria (*Prevotellaceae* and *Rikenellaceae*), probiotics (*Bifidobacteriaceae* and *Lactobacillaceae*), and mucin-degrading bacteria (*Akkermansia* and *Helicobacter*) and decreased the abundance of *Desulfovibrionaceae* and harmful metabolites in normal mice. Recently, Xiao Chen et al. (2020) found that 6 weeks of lactulose intervention altered gut microbiota, increased SCFA levels, and ameliorated bone loss induced by lack of estrogen.

However, the effect of a low concentration of lactulose on stroke, including whether it can modulate gut dysbiosis and metabolic disorders and help improve outcomes after stroke, is unknown. Although several studies have associated lactulose with microbiota or metabolites, the direct effects of lactulose on stroke outcomes have not been determined. An understanding of how lactulose contributes to stroke outcomes may enable its use as a therapeutic target. In this study, we tested the hypothesis that 15% lactulose could improve stroke outcomes by examining inflammatory factor expression and fecal flora and plasma metabolite composition using omics technologies. Overall, we found that lactulose had an anti-inflammatory effect on both the brain and gut and partially corrected metabolic disorders and dysbiosis. The current data support a positive effect of lactulose on stroke outcomes.

## Materials and Methods

### Experimental Design

Six to eight-week-old male C57BL/6 mice (20–25 g) were purchased from HFK Bioscience Corporation (Beijing, China). In the experimental period, mice were allowed to eat and drink freely in a room with a natural light cycle. All mice were randomly divided into three groups: healthy control (HC), photothrombotic stroke + triple-distilled water (PTS_TDW), and photothrombotic stroke + lactulose (PTS_LAC, Yuanye Bio-Technology Co., Ltd, Shanghai, China, with concentration of 15%, 150 μL per day.). A concentration of 15% lactulose was used because previous studies (Mao et al., 2016; Zhang et al., 2019b) indicated that this concentration was the most suitable for exhibiting prebiotic and anti-inflammatory effects. All experiments in this study were approved by the Tianjin Medical University General Hospital Animal Care and Use Committee. After one week of adaptation to the new environment, mice in the PTS_TDW and PTS_LAC groups were subjected to photothrombotic stroke modeling. Twenty-four hours after stroke, mice in these groups were treated with triple distilled water or lactulose, respectively, by oral gavage for 14 consecutive days. Eleven mice per group were used for neurological function tests and weight recording, 6 mice per group were used for flow cytometry and omics-related analysis, and 5 mice per group were used for qPCR and Elisa test.

### Photothrombotic Stroke Model

This method has been described previously (Yan et al., 2020). Briefly, after anesthetization with 5% chloral hydrate by intraperitoneal injection, the scalp area was shaved and disinfected with iodophor, and then bregma was exposed. Ten minutes after intraperitoneal injection with Rose Bengal dye (10 mg/mL), mice were subjected to 20 minutes of illumination with a fiber-optic bundle of a cold light source. Finally, the incision was sutured and disinfected again.

### Neurological Function Tests and Infarct Volume Measurement

A series of neurological functional tests (Yan et al., 2020) including determination of the Modified Neurological Severity Score (mNSS) and the foot-fault test were performed prior to stroke and on days 1, 3, 7, and 14 after stroke by an experimenter who was blinded to the study groups. The mNSS test, ranging from 0 to 18 points, mainly includes motor, sensory, balance, and reflex tests (6 points for motor function, 2 points for sensory function, 6 points for the balance beam test, and 4 points for reflex activities). The foot-fault test was used to evaluate the contralateral motor function deficits. Mice were photographed walking on an irregular grid for a period of time in a quiet environment. The contralateral limb foot faults percentage was determined. A higher score on these two tests indicated a more severe neurological deficit.

Paraffin block from brain was prepared and cut into 8-μm sections. Five coronal sections obtained from the lesion underwent HE staining. The percentage of lesion compared with the contralateral hemisphere was calculated for infarct volume measurement.

Body weight, which reflects the general physical condition of mice and stroke outcome and recovery, was recorded on days 0, 1, 3, 5, 7, and 14.

### Quantitative Real-Time PCR

Briefly, total RNA was isolated from the brain and cecum with TRIzol reagent (Invitrogen, CA, USA) at 14 days after stroke. Then, RNA was quantified and reverse transcribed to cDNA using a cDNA Synthesis Kit (Transgen, Beijing, China). PCR reactions were performed with a CFX96 real-time PCR system (BioRad, Hercules, CA, USA). Relative gene expression was calculated using the 2−ΔΔCT method. All primer sequences used are shown below:

GAPDH (Gene ID: 14433):

Forward: GCCAAGGCTGTGGGCAAGGT; Reverse: TCTCCAGGCGGCACGTCAGA

MCP-1 (Gene ID: 20296):

Forward: CTGCTACTCATTCACCAGCAAG; Reverse: CTCTCTCTTGAGCTTGGTGACA

IL-17a (Gene ID: 16171):

Forward: TTTAACTCCCTTGGCGCAAAA; Reverse: CTTTCCCTCCGCATTGACAC

TNFα (Gene ID: 21926):

Forward: TACTCCCAGGTTCTCTTCAAGG; Reverse: GGAGGTTGACTTTCTCCTGGTA

IL-1β (Gene ID: 16176):

Forward: TCCAGGATGAGGACATGAGCAC; Reverse: GAACGTCACACACCAGCAGGTTA

Muc2 (Gene ID: 17831):

Forward: ACGTGTCATATTTGCACCTCT; Reverse: TCAACATTGAGAGTGCCAACT

TLR4 (Gene ID: 21898):

Forward: AGTCAGAATGAGGACTGGGTGAG; Reverse: GTAGTGAAGGCAGAGGTGAAAGC

TGFβ (Gene ID: 21803):

Forward: TGCGCTTGCAGAGATTAAAA; Reverse: CGTCAAAAGACAGCCACTCA

Nrf2 (Gene ID: 18024):

Forward: GGACATGGAGCAAGTTTGGC; Reverse: CCAGCGAGGAGATCGATGAG

Claudin1 (Gene ID: 12737):

Forward: TGTGTCCACCATTGGCATGA; Reverse: CTGGCATTGATGGGGGTCAA

Occludin (Gene ID: 18260):

Forward: TGGCAAAGTGAATGGCAAGC; Reverse: TCATAGTGGTCAGGGTCCGT

### Flow Cytometry Analysis

Mice were sacrificed at 14 days after stroke and brain harvested and single-cell suspension prepared. In short, each brain was minced with eye scissors then digested in collagenase IV for 60 minutes. Next, after resuspended in 30% Percoll and centrifugation, the single-cell suspension of the brain was tested by flow cytometry (BD FACSAria, BD Biosciences, San Jose, CA, USA) and analyzed with FlowJo software. Antibodies specific to mouse CD45, CD11b, Ly6G, and F4/80 were used (BioLegend, Inc., San Diego, CA, USA).

### Elisa

Plasma was obtained by extracting eyeballs, centrifuging (3000rpm, 10 min). 10μL/well was used in three replicates wells to run IL-17a and LPS Elisa (Beijing Gersion Bio-Technology Co., Ltd., Beijing, China) following standard protocol.

### 16S rDNA Amplicon Sequencing

Fecal genomic DNA was extracted according to the cetyltrimethylammonium bromide/sodium dodecyl sulfate method. DNA was diluted after the concentration and purity were determined. Then, using specific primers, the V3 and V4 variable regions of 16S rDNA were amplified. After PCR reactions were performed, the PCR products were quantified, qualified, mixed, and purified. Then, sequencing libraries were constructed using the TruSeq^®^ DNA PCR-Free Sample Preparation Kit (Illumina, San Diego, CA, USA). Finally, the constructed libraries were sequenced using an Illumina HiSeq 2500 instrument. The raw data were deposited in the NCBI-SRA database with the accession number SRP298849 (https://www.ncbi.nlm.nih.gov/bioproject/PRJNA686830).

### Determination of SCFAs in Feces

Fecal samples were carefully thawed on ice. Then, 30 mg of each sample was added to a centrifuge tube, and 0.5% phosphoric acid, ethyl acetate, and 4-methyl valeric acid were sequentially added and homogenized while supernatants were extracted. An Agilent Model 7890A/5975C gas chromatography-mass spectrometry system (Agilent, Santa Clara, CA, USA) was used for gas chromatography-mass spectrometry analysis, and an MSD ChemStation (Santa Clara, CA, USA) was used to process data to quantify SCFAs.

### Untargeted Metabolomics Analysis

Prior to sacrificing the mice, plasma was collected for metabolomics analysis. An ultra-high-performance liquid chromatography device (Agilent 1290 Infinity LC) coupled with an AB Triple TOF 6600 was used for liquid chromatography tandem mass spectrometry analysis. Multi-dimensional and univariate statistical analyses were performed after raw data were processed.

### Correlations

Differentially abundant genera and metabolites were scaled according to the Z-score and concatenated into one matrix. The Pearson algorithm in R Version 3.5.1 was used to calculate the correlation coefficients among all molecules in a matrix because raw data were non‐normally distributed.

### Statistical Analysis

Normal data were analyzed with GraphPad Prism 8.0 and are presented as the mean ± SEM. Two-way repeated measures ANOVA with Sidak’s multiple comparisons test and one-way ANOVA were performed.

## Results

### Lactulose Supplementation Significantly Improved Long-Term Functional Outcomes and Did Not Affect Body Weight After Stroke in Mice

**Figure 1:**
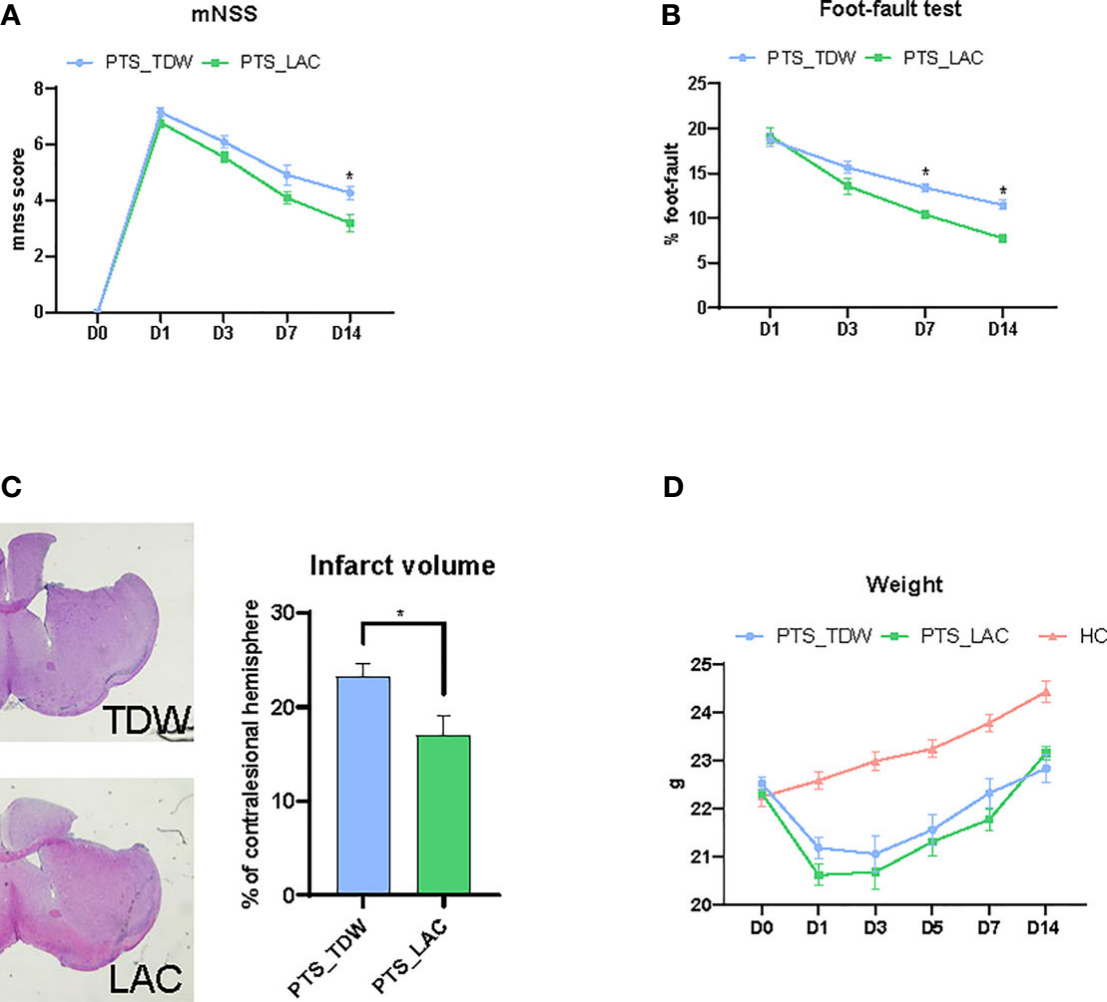
**(A–C)** Lactulose supplementation significantly improved functional outcomes (n = 11/group, two-way repeated measures ANOVA with Sidak’s multiple comparisons test, *p < 0.05) and decreased infarct volume (n = 6/group, unpaired 2-tailed Student’s t-test., *p < 0.05) **(D)** Lactulose supplementation did not affect the body weight of mice (n = 11/group, two-way repeated measures ANOVA with Sidak’s multiple comparisons test, *p < 0.05).

To evaluate the therapeutic effects of lactulose supplementation in mice with stroke, modified neurological severity score and foot-fault tests were used to evaluate neurological function at 24 hours after stroke. **Figure 1A** shows that lactulose supplementation in mice with stroke significantly decreased the modified neurological severity score at 14 days after stroke. Furthermore, lactulose significantly decreased foot-fault test scores as early as 7 days after stroke as shown in **Figure 1B**. We further calculated infarct volume, and the results showed that lactulose supplementation significantly decreased lesion volumn (**Figure 1C**).

The body weight, which was used to determine the general physical condition of mice, was recorded on days 0, 1, 3, 5, 7, and 14. Mice in the PTS_TDW and PTS_LAC groups showed significant loss of body weight after the operation on day 1. No significant differences were found between these two groups at any time points (**Figure 1D**).

### Lactulose Supplementation Significantly Decreased Inflammatory Reaction in the Brain

Localized inflammation, or even global brain inflammation (Shi et al., 2019), occurs after stroke onset. Cascades of inflammatory mediators such as cytokines and chemokines are produced, accompanied by leukocyte invasion. Peripheral leukocytes including monocytes/macrophages and neutrophils, infiltrate into the ischemic peripheral area, further aggravating stroke damage. Among the inflammatory mediators, interleukin (IL)-1β, tumor necrosis factor α (TNFα) and monocyte chemoattractant protein-1 (MCP-1) are classic factors that have been found in both experimental models and human stroke. Brain damage or inflammation caused by ischemic injury can activate TLR4, an important member of the TLR protein family (Caso et al., 2007). Transforming growth factor-β (TGFβ) is an anti-inflammatory and neuroprotective cytokine (Cekanaviciute et al., 2014). Nuclear factor erythroid 2-related factor 2 (Nrf2) also plays an important role in oxidative stress resistance and anti-inflammation responses, and it is a potential therapeutic target for central nervous system diseases, especially for ischemic stroke (Liu et al., 2019).

**Figure 2:**
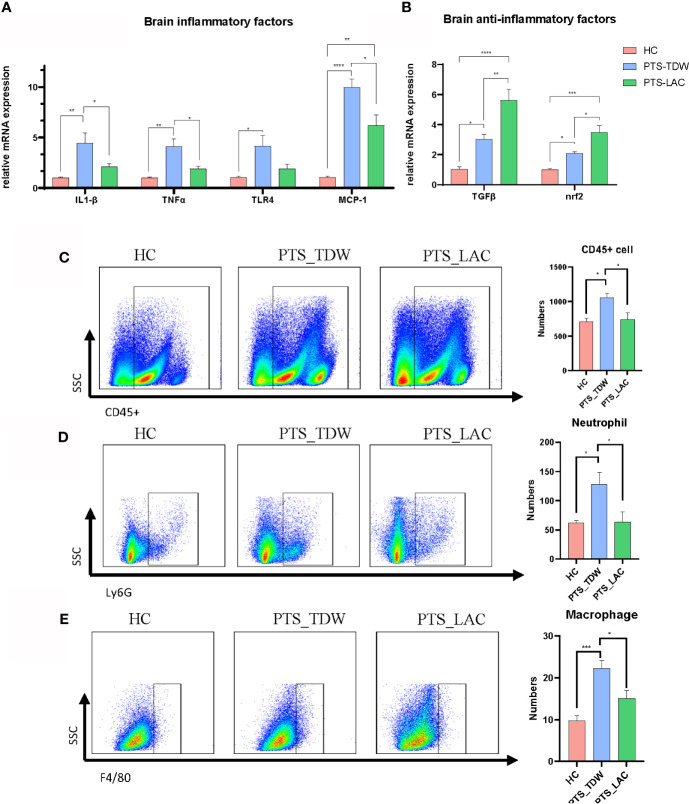
**(A, B)** Lactulose supplementation significantly decreased inflammatory factor expression and increased anti-inflammatory factor expression and antioxidative regulators in the brain (n=5/group, one-way ANOVA with Tukey’s multiple comparisons test, *p < 0.05) at 14 days after stroke. **(C–E)** Lactulose supplementation significantly decreased the number of CD45^+^cell, CD45^+^CD11b^+^Ly6G^+^ neutrophil and CD45^+^CD11b^+^F4/80^+^macrophage in the brain (n = 6/group, one-way ANOVA with Tukey’s multiple comparisons test, *p < 0.05) at 14 days after stroke. **p < 0.01, ***p < 0.001, ****p < 0.0001.

In this study, we found that the brain expression levels of IL-1β, TNFα, MCP-1, and TLR4 in the PTS_TDW group were significantly elevated, even at 14 days after stroke, compared with the HC group; while lactulose supplementation significantly decreased the expression of these factors, as shown in **Figures 2A, B**. Furthermore, lactulose supplementation remarkably increased TGFβ and Nrf2 expression.

Flow cytometry was then used to analyze the numbers of inflammatory cells in the brain among the three groups (the gate strategy is shown in **Supplemental Figure 1**). CD45 is expressed on leukocyte and microglia, CD45, CD11b and Ly6G are co-expressed on neutrophil, and CD45, CD11b and F4/80 are co-expressed on macrophage. We found that the number of CD45^+^cell, CD45^+^CD11b^+^Ly6G^+^ neutrophil, and CD45^+^CD11b^+^F4/80^+^macrophage were significantly higher in the PTS_TDW group compared with the HC group, and the number of these three types of cells significantly decreased in the PTS_LAC group (**Figures 2C–E**). These data suggest that lactulose administration could reduce the inflammatory reaction by prohibiting inflammatory factors production and inflammatory cell infiltration.

### Lactulose Supplementation Decreased the Inflammatory Reaction in the Gut and Restored Intestinal Barrier Injury After Stroke

In addition to the brain, stroke can also cause inflammation in other locations, such as the gut and blood (Spychala et al., 2018; Blasco et al., 2020). Many studies have found that inflammatory mediators are elevated after stroke, including IL-17a (Waisman et al., 2015).

**Figure 3:**
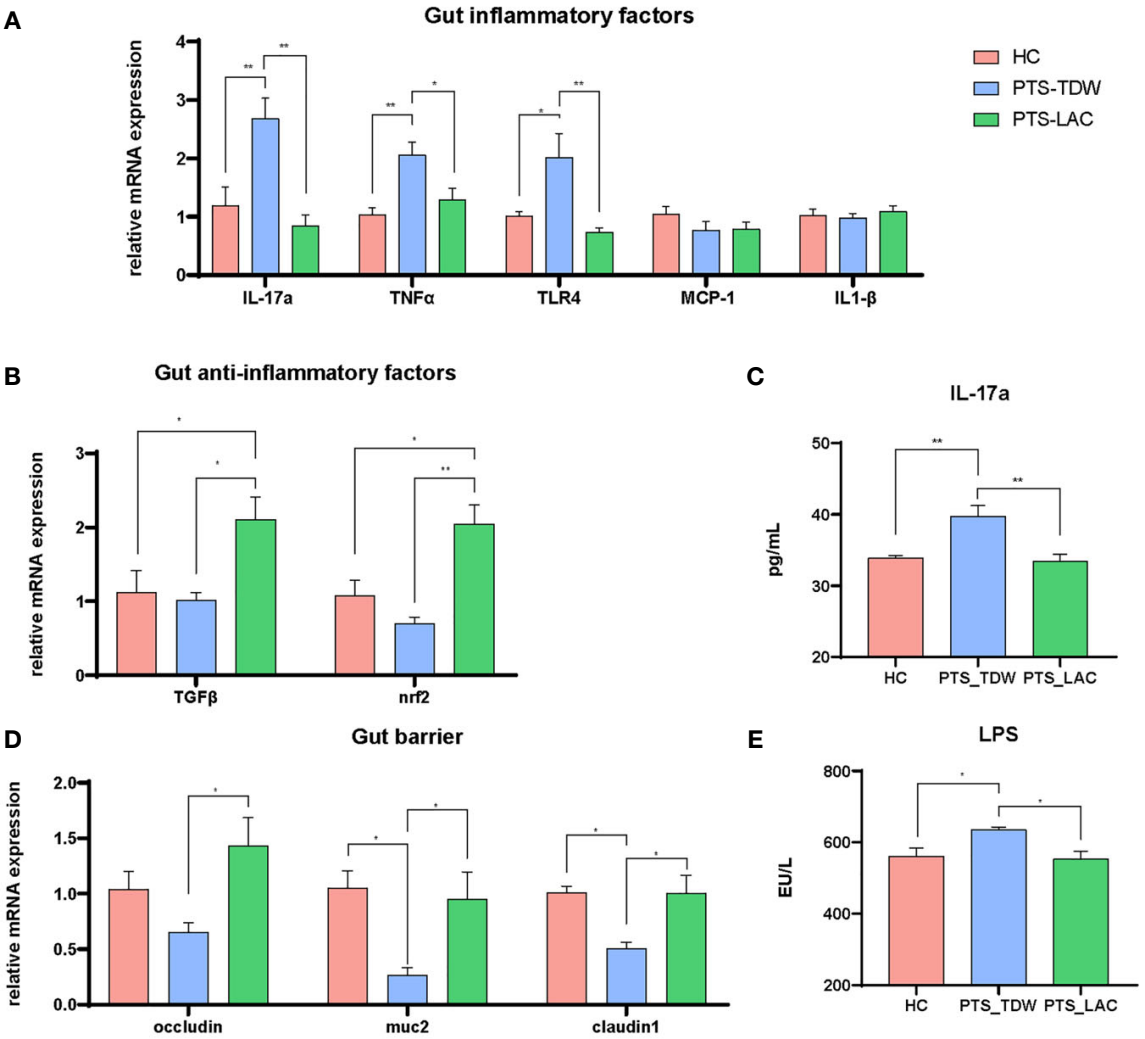
**(A, B)** Lactulose supplementation significantly decreased inflammatory factor expression and increased anti-inflammatory factor expression and antioxidative regulators in the gut **(C)** Lactulose supplementation significantly decreased IL-17a level in the plasma. **(D, E)** Lactulose supplementation affected the intestinal barrier and muc2 expression (n = 5/group, one-way ANOVA with Tukey’s multiple comparisons test, *p < 0.05) at 14 days after stroke. **p < 0.01.

In contrast to the brain, stroke markedly increased expression of inflammatory-related factors, including IL-17a, TNFα, and TLR4, in the gut (**Figure 3A**); however, IL-1β and MCP-1 levels were not increased as in the brain. After lactulose intervention, IL-17a, TNFα, and TLR4 were suppressed, in addition, TGFβ and Nrf2 were also activated (**Figure 3B**). We then examined the IL-17a level in the blood. The results showed that the level of IL-17a in the PTS_TDW group was significantly higher than that in the HC group, while the IL-17a level in the PTS_LAC group was significantly lower than that in the PTS_TDW group (**Figure 3C**).

The gut barrier is composed of a mucus layer, epithelial cells, intercellular junctions, and immune cells. The decreased expression levels of claudin-1, muc2, and occludin and the increased level of LPS indicated disruption of the intestinal barrier. Claudin-1, muc2, and occludin were significantly increased and LPS was significantly decreased after lactulose supplementation (**Figures 3D, E**), indicating that lactulose might help to restore and repair the intestinal barrier.

### Lactulose Partially Restored Gut Microbiota Dysbiosis After Stroke

**Figure 4:**
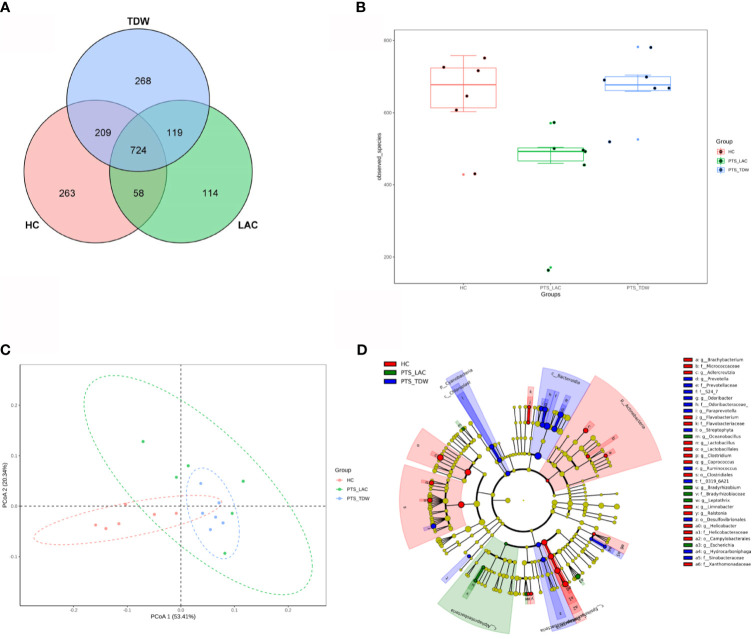
Variations in microbiota among the three groups. **(A)** Venn diagram of the operational taxonomic units among the three groups. **(B)** Boxplots of α-diversity as measured by observed species (n = 6). **(C)** Variations in microbiota among the three groups according to principal coordinates analysis. **(D)** A cladogram of the linear discriminant analysis effect size shows significantly different taxa among the three groups from the phylum to family level.

Feces were collected to investigate the gut microbiota in the three groups, and 16S rDNA sequencing was performed. Shared and distinct operational taxonomic units among the three groups are shown in a Venn diagram (**Figure 4A**). In total, 724 operational taxonomic units were shared among all groups. The α-diversity, including the observed species and ACE, Chao1, and Shannon indices, was used to analyze microbial diversity within the community, while the β-diversity was used to analyze diversity among different communities. In this study, lactulose significantly altered α-diversity because the diversity of the PTS_LAC group was lower than that of the PTS_TDW group (**Figure 4B**); however, after 14 days, there were no significant differences between the PTS_TDW and HC groups in this study. Principal coordinates analysis using the weighted UniFrac distances (**Figure 4C**) showed that all three groups can be clustered. A cladogram of the linear discriminant analysis effect size showed significantly different taxa among the three groups (**Figure 4D**).

**Figure 5:**
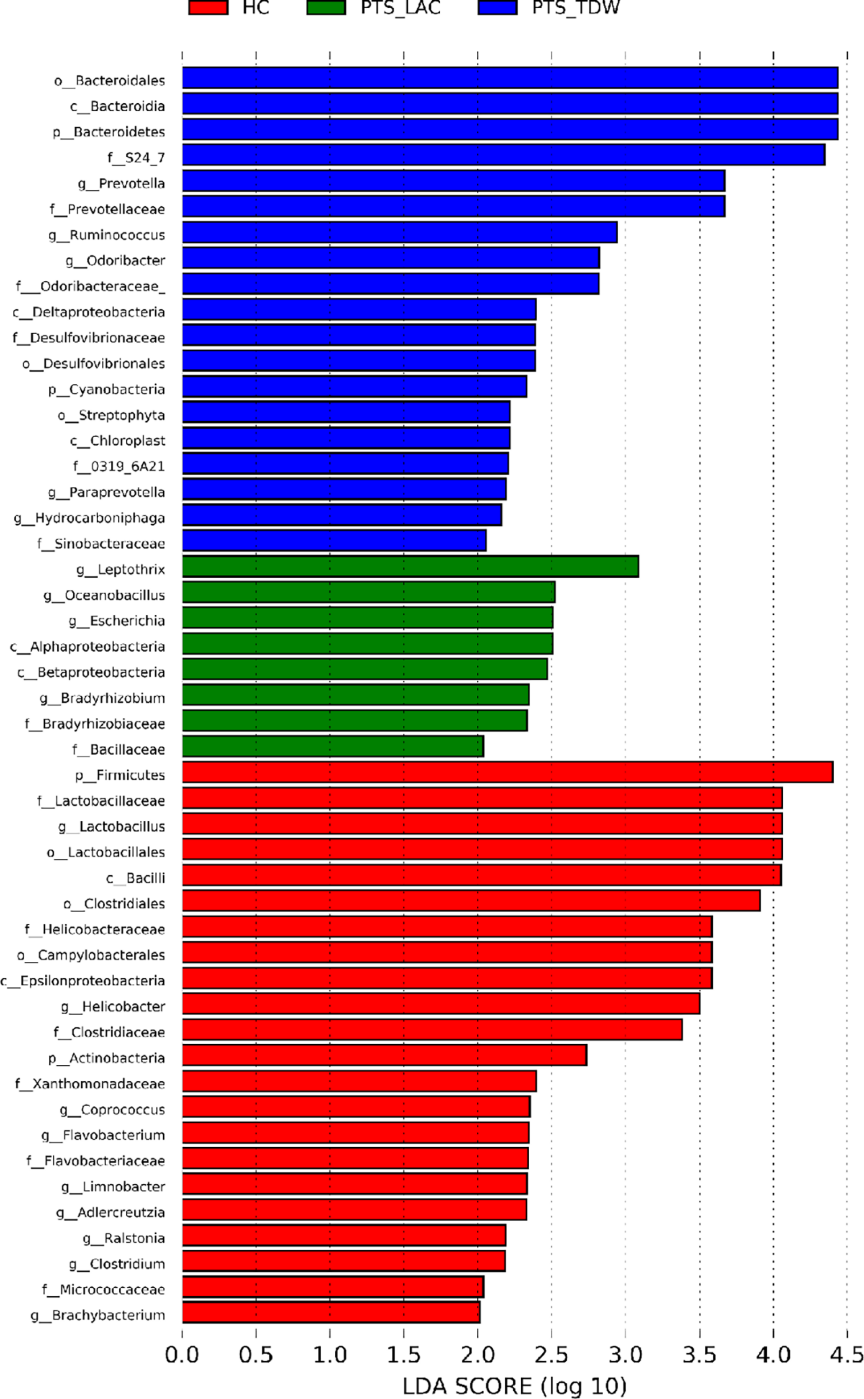
Linear discriminant analysis (LDA) was used to analyze biomarker taxa (LDA scores≥2) in the three groups.

The linear discriminant analysis effect size was used to analyze bacterial genera specific to each group, and biomarker taxa with a linear discriminant analysis value ≥2 are shown in **Figure 5**. The results showed that, at the phylum level, *Firmicutes* and *Actinobacteria* were more abundant in the HC group, while *Bacteroidetes* and *Cyanobacteria* were more abundant in the PTS_TDW group. At the family level, *Lactobacillaceae, Clostridiaceae, Helicobacteraceae, Micrococcaceae*, and *Flavobacteriaceae* were more abundant in the HC group; *Desulfovibrionaceae, Odoribacteraceae, Prevotellaceae*, and *Sinobacteraceae* were more abundant in the PTS_TDW group; and *Bradyrhizobiaceae* were more abundant in the PTS_LAC group. At the genus level, *Lactobacillus*, *Clostridium, Flavobacterium, Brachybacterium*, and *Helicobacter* were more abundant in the HC group; *Ruminococcus*, *Prevotella*, *Paraprevotella*, and *Odoribacter* were more abundant in the PTS_TDW group; and *Bradyrhizobium*, *Oceanobacillus*, *Escherichia*, and *Leptothrix* were more abundant in the PTS_LAC group.

Kyoto Encyclopedia of Genes and Genomes (KEGG) pathway analyses were performed to predict functions according to the species composition. Compared with the HC group, pathways including Endocrine System, Glycan Biosynthesis and Metabolism, Excretory System, Biosynthesis of Other Secondary Metabolites, Transport and Catabolism, Amino Acid Metabolism, Metabolism of Cofactors and Vitamins, Metabolism of Other Amino Acids, and Digestive System were highly expressed in the PTS_TDW group. Compared with the PTS_TDW group, pathways including Cardiovascular Diseases, Poorly Characterized, and Nervous System were highly expressed in the PTS_LAC group.

### Lactulose Regulated Metabolomic Changes in the Plasma of Mice With Photothrombotic Stroke

The plasma metabolome may play an important role in the link among the gut, gut microbiota, and brain. Therefore, we performed an untargeted metabolome profiling analysis by ultra-high performance liquid chromatography-quadrupole time-of-flight mass spectrometry to explore the impact of lactulose on metabolic changes in the plasma. We also examined the fecal SCFA level among three groups, and the results are shown in the **Supplemental Materials**.

A total of 64 significant differential metabolites and 46 altered KEGG pathways were identified between the PTS_TDW and HC group, 36 significant differential metabolites and 17 altered KEGG pathways were identified between the PTS_LAC and PTS_TDW groups, and 35 significant differential metabolites and 45 altered KEGG pathways were identified between the PTS_LAC and HC groups.

**Figure 6:**
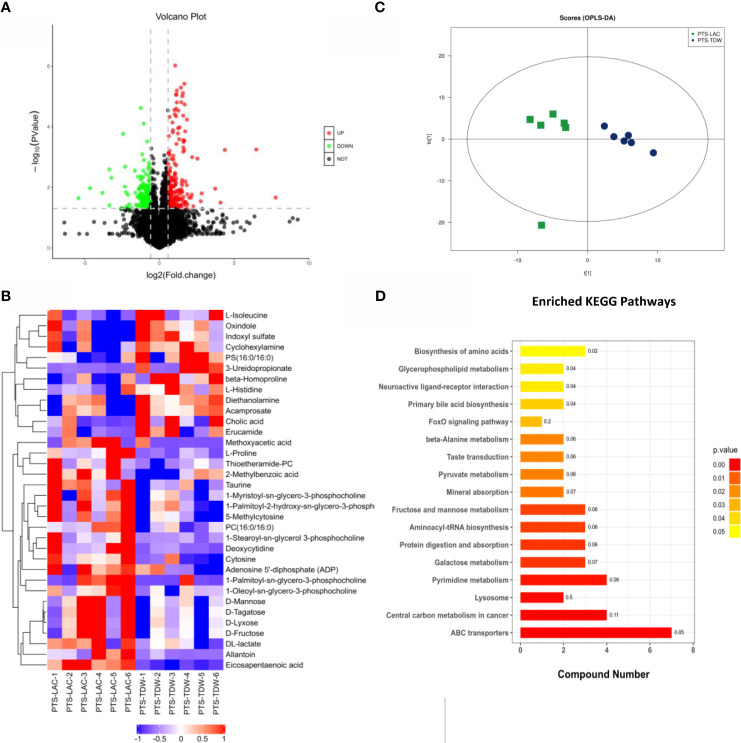
**(A)** Volcano plot for the PTS_LAC and PTS_TDW groups in the positive mode. Red and green dots indicate significant differential metabolites with fold changes >1.5 and p values <0.05. **(B)** Heatmap showing normalized values of 34 metabolites that were differentially abundant among the PTS_LAC and PTS_TDW groups. **(C)** Orthogonal partial least squares discrimination analysis: score plots of the PTS_LAC and PTS_TDW groups in the positive ion mode. **(D)** Kyoto Encyclopedia of Genes and Genomes pathways enriched in the PTS_LAC group compared with the PTS_TDW group.

In order to explore specific differences after lactulose intervention, a volcano plot (**Figure 6A**) based on a univariate analysis and a hierarchical clustering graph (**Figure 6B**) were generated. The orthogonal partial least squares discrimination analysis score plots (**Figure 6C**) of positive modes showed significant dispersion of the two groups.

In addition, KEGG pathway enrichment analysis of differentially expressed metabolites between the PTS_TDW and PTS_LAC groups was performed using the Fisher exact test (**Figure 6D**). The results showed that a total of 17 pathways, including ABC transporters, Central carbon metabolism in cancer, Lysosome, Pyrimidine metabolism, Galactose metabolism, Protein digestion and absorption, Aminoacyl-tRNA biosynthesis, Fructose and mannose metabolism, Mineral absorption, Pyruvate metabolism, Taste transduction, β-Alanine metabolism, FoxO signaling pathway, Primary bile acid biosynthesis, Neuroactive ligand-receptor interaction, Glycerophospholipid metabolism, and Biosynthesis of amino acids, were significantly altered, and ABC transporters were highly altered.

Spearman correlation analysis was performed to further understand the correlation between different metabolites in the PTS_LAC and PTS_TDW groups. D-mannose was positively correlated with DL-lactate (+0.85), D-lyxose (+0.99), D-tagatose (+0.99), and D-fructose (+0.99). Allantoin was negatively correlated with indoxyl sulfate (IS) (−0.69). L-proline was positively correlated with deoxycytidine (0.84) and negatively correlated with diethanolamine (−0.77) and L-histidine (−0.76).

### Correlation Analysis Between Plasma Metabolites and Fecal Microbiota Composition

**Figure 7:**
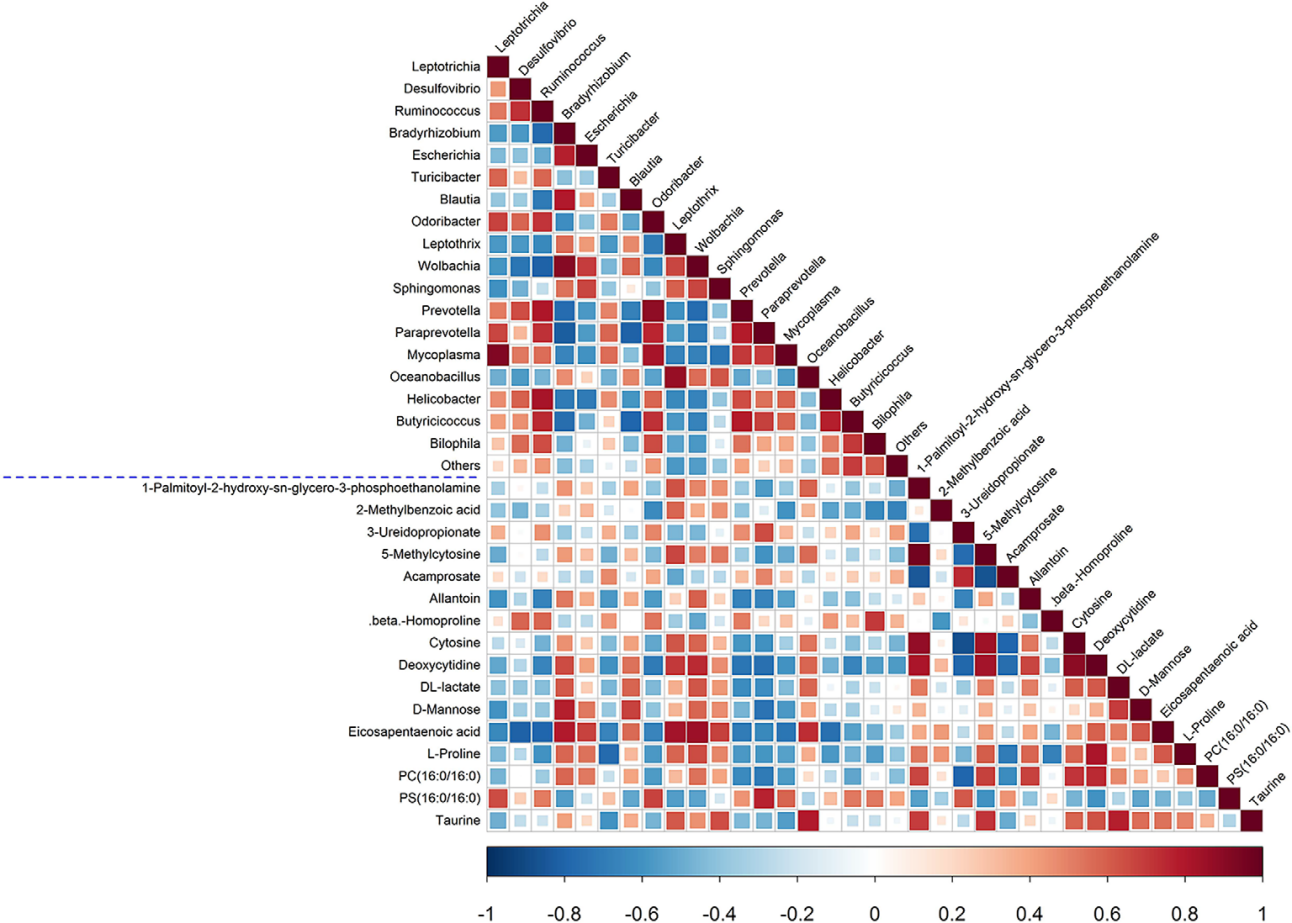
Spearman correlation analysis of fecal microbiota and plasma metabolites. The p-values are depicted in blue and red, where red represents a positive correlation and blue represents a negative correlation.

Next, a correlation analysis was performed to investigate the association between plasma metabolites and the fecal microbiota composition, and the results are illustrated in a heatmap (**Figure 7**). There were several findings of note. First, the levels of IS and nicotinamide N-oxide were negatively correlated with Lactobacillus (−0.78 and −0.82), and IS was positively correlated with oxindole (+0.9). Second, the levels of eicosapentaenoic acid (EPA) and taurine were positively correlated with Oceanobacillus (+0.74 and +0.8), and EPA was also positively correlated with Bradyrhizobium (+0.8), Leptothrix (+0.83), and Wolbachia (+0.86) and negatively correlated with Desulfovibrio (−0.8), Ruminococcus (−0.79), and Helicobacter (−0.81). Third, the allantoin level was positively correlated with Candidatus Phlomobacter (+0.96) and Leptothrix (+0.76) and negatively correlated with Leptotrichia (−0.75) and Fusobacterium (−0.77). Fourth, D-mannose was positively correlated with Bradyrhizobium (+0.77) and negatively correlated with Acamprosate (−0.77) and Benzylazanium (−0.78).

Hierarchical clustering was performed to investigate the varying trends of metabolites among the three groups. Four expression patterns (profile 0–3) were obtained in both positive and negative modes (**Supplemental Figure 3**). The x-axis indicates the different groups (HC, PTS_TDW, and PTS_LAC groups), and the y-axis indicates the standardized levels of metabolites. Among all patterns, patterns 1 and 2, which reflect metabolites fluctuating up and down, attracted our attention.

In the positive mode, pattern 1 included D-mannose, L-proline, citrate, uracil, 2’-deoxyuridine, L-leucine, thiamine, triethanolamine, and pantothenate, and pattern 2 included IS, ADP, L-glutamine, nicotinamide N-oxide, 2-methylbutyroylcarnitine, and 3-ureidopropionate. In the negative mode, pattern 1 included allantoin, taurine, DL-lactate, L-galactono-1,4-lactone, 3-hydroxydodecanoic acid, and 2-methyl-3-hydroxybutyric acid, and pattern 2 included acamprosate.

## Discussion

Our data showed that lactulose improved neurological function, suppressed inflammation in the brain and gut, regulated metabolic disturbance, and inhibited harmful bacteria in mice after stroke.

Lactulose downregulated inflammatory mediators and upregulated the expression of anti-inflammatory factors such as TGFβ and Nrf2 in both the brain and gut. Initiation of inflammation after stroke worsens the functional prognosis, while treatments that target inflammatory cytokines, such as TNFα and IL-1, have been shown to be effective (Lambertsen et al., 2019) in restoring neurological outcomes after stroke. In this study, administration of lactulose not only suppressed inflammation in the brain and gut but also promoted anti-inflammatory factors such as TGFβ and Nrf2, which is consistent with a previous study (Zhai et al., 2013). The effects of lactulose on different organs were mediated by various inflammatory pathways/mechanisms.

Furthermore, we found that lactulose significantly upregulated gut barrier markers including claudin-1, muc2, and occludin. These results provided evidence that lactulose might restore gut barrier damage after stroke. In addition, stroke leads to gut dysbiosis and activates the immune system, which aggravates the neurological outcome after stroke (Benakis et al., 2016; Durgan et al., 2019).

In this study, no significant difference in α-diversity was observed between the HC group and the PTS_TDW group. However, there is no uniform conclusion in published studies regarding the change in α-diversity after stroke (Singh et al., 2016; Li et al., 2019). Compared with the HC group, the abundance of probiotics such as *Lactobacillus* decreased, and pathogens such as *Neisseria* and *Fusobacterium* increased in the PTS_TDW group. Compared with the PTS_TDW group, lactulose supplementation decreased the abundance of pro-inflammatory taxa (Gao et al., 2018; Ma et al., 2018) such as *Desulfovibrio, Helicobacter*, and *Turicibacter*, which might partially explain the decrease in α-diversity after drug administration [The relative abundance genus csv file and the heatmap of clustering for genus abundance and are in **Supplemental Material** and **Supplemental Figure 4**)]. In addition, the ratio of *Firmicutes* to *Bacteroidetes*, which is seen as a marker of dysbiosis in some studies, was decreased in the PTS_TDW group compared with the HC group (the average F/B ratios of the HC, PTS_TDW and PTS_LAC groups were 1.186,0.331 and 0.460 respectively; the relative abundance phylum csv file and the heatmap of clustering for phylum abundance are shown in the **Supplemental Material** and **Supplemental Figure 5**), while another study found that the *Firmicutes* to *Bacteroidetes* ratio increased after stroke (Brichacek et al., 2020); therefore, we speculated that an altered *Firmicutes* to *Bacteroidetes* ratio indicated gut dysbiosis.

Our results showed that Desulfovibrionaceae, one harmful bacterium, decreased after lactulose administration, while there is no significant difference was observed for *Bifidobacterium* and *Lactobacillus*, which is similar to previous studies (Zhai et al., 2018; Zhang et al., 2019a), although Lactulose has long been viewed as a bifidus factor.

Our data showed that lactulose treatment could decrease accumulation of some harmful metabolites, such as IS, and increase the levels of some beneficial metabolites in plasma after stroke. IS, which is a toxic uremic solute derived from tryptophan metabolism, has been widely studied in renal disease, especially chronic kidney disease (Vanholder et al., 2014). In addition to the renal system, IS affects the cardiovascular system and central nervous system (Gao and Liu, 2017; Hung et al., 2017). Many studies have found that IS promotes inflammation, oxidative damage, and fibrosis and induces gut barrier (Huang et al., 2020) and endothelial cell dysfunction. Our study showed that IS was elevated after stroke, and lactulose significantly decreased its accumulation. Recently, a study found that stroke may induce kidney dysfunction (Zhao et al., 2020); therefore, IS accumulation may be a potential mechanism of stroke-induced kidney dysfunction.

**Figure 8:**
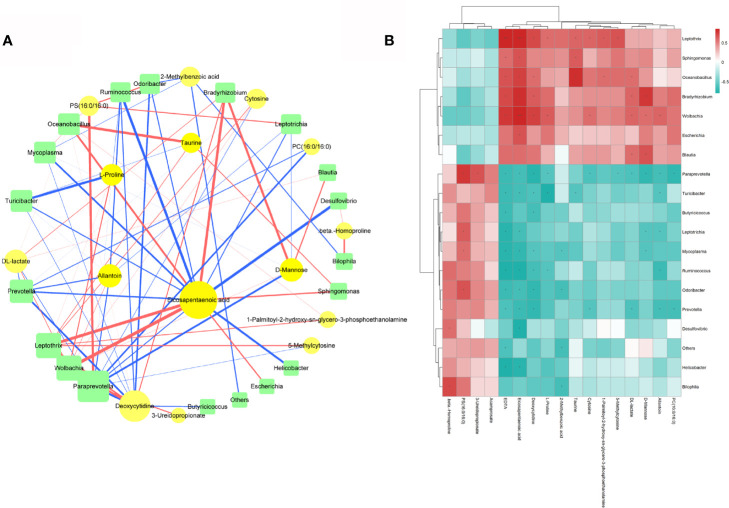
**(A)** Spearman Correlation network among the differentially expressed fecal microbiota and plasma metabolites; circles represent metabolites and squares represent microbiota. Different line colors represent positive (red) and negative (blue) correlation coefficients, and the line width is proportional to the absolute value of the correlation coefficient. **(B)** Hierarchical clustering heatmap of differentially expressed fecal microbiota and plasma metabolites.

The beneficial metabolites induced by lactulose supplementation, which were correlated with some taxa, included EPA, allantoin, taurine, and D-mannose. A network and a hierarchical clustering heatmap were generated to intuitively exhibit correlations among the differentially expressed fecal microbiota and plasma metabolites (**Figures 8A, B**).

EPA, an omega-3 polyunsaturated fatty acid, exerts cardiovascular protective effects *via* its anti-inflammation and antioxidative stress activities, inhibition of platelet activity, and ability to decrease plasma triglyceride levels (Innes and Calder, 2018). The presence of EPA in the network was striking because it was connected with many florae. Many studies (Nakase et al., 2015; Aung et al., 2018; Alvarez Campano et al., 2019) have shown that EPA supplementation can improve the prognosis of patients and prevent cardiovascular and cerebrovascular diseases. Therefore, increased EPA levels might lead to good outcomes after stroke. In most mammals, other than humans, uric acid is quickly degraded to allantoin by urate oxidase in purine metabolism (Maiuolo et al., 2016). Uric acid is also seen as a promising biomarker to reflect the oxidative status (Seet et al., 2011). Studies have found that uric acid therapy is effective for ischemic stroke treatment (Kand’ár et al., 2006; Llull et al., 2015). However, no stroke-related study has shown that allantoin has a similar effect to uric acid, but allantoin therapy has been used to treat other diseases, such as gastric ulcers (da Silva et al., 2018) and gastritis (Eslami-Farsani et al., 2018), which may offer a novel therapeutic opportunity for stroke. Therefore, further study is urgently needed. Taurine has cytoprotective, antioxidative stress, anti-inflammatory, and barrier integrity-maintaining effects (Schaffer and Kim, 2018; Jakaria et al., 2019). In animal experiments and clinical trials, taurine has been used to treat neurological (Hou et al., 2018; Ohsawa et al., 2019), cardiovascular (Katakawa et al., 2016), and metabolic diseases (Obrosova et al., 2001), especially stroke (Guan et al., 2011; Sun et al., 2012; Jin et al., 2018). Our study found that taurine was decreased after stroke and increased by lactulose, which perhaps could explain the good neurological performance of the PTS_LAC group. D-mannose is also a beneficial metabolite. Dunfang Zhang et al. (2017) found that D-mannose activated TGFβ expression, promoted T regulatory cell differentiation, and inhibited inflammation. The activation of TGFβ expression in both the brain and gut found in our study may be related to the increase in D-mannose.

Therefore, we speculated that an increase in beneficial metabolites and a decrease in harmful metabolites may have led to improved neurological outcomes after stroke. To our knowledge, this is the first study to examine the effects of lactulose on stroke outcomes using omics technologies (16S sequencing and metabolomics). However, inclusion of a group to explore how lactulose affects healthy mice and extension of the duration of lactulose administration would strengthen our results.

## Conclusions

In summary, ischemic stroke led to inflammatory reactions in both the brain and gut, resulting in gut barrier disruption and dysbiosis, which could be partly alleviated by lactulose. The effects of lactulose may be attributable to repressing harmful bacteria and metabolic disorder, repairing gut barrier disruption, and inhibiting inflammatory reaction after stroke in mice.

## Data Availability Statement

The raw data has been deposited and made public in the NCBI-SRA database with the accession number SRP298849.

## Ethics Statement

The animal study was reviewed and approved by Tianjin Medical University General Hospital Animal Care and Use Committee.

## Author Contributions

TY: experimental design and gave final approval of manuscript. QY: experimental design, wrote the manuscript, performed experiments, analyzed data and prepared figures. LX, SH, YS, RW, XL, JW, and RL: performed experiments, analyzed data and prepared figures. All authors contributed to the article and approved the submitted version.

## Funding

This work was supported by the National Natural Science Foundation of China, Grant Numbers: 81671144, 91746205.

## Conflict of Interest

The authors declare that the research was conducted in the absence of any commercial or financial relationships that could be construed as a potential conflict of interest.

## References

[B1] Alvarez Campano C. G. Macleod M. J. Aucott L. Thies F. (2019). Marine-Derived N-3 Fatty Acids Therapy for Stroke. Cochrane Database Syst. Rev. 6 (6), Cd012815. 10.1002/14651858.CD012815.pub2 31242320 PMC6594574

[B2] Anderson J. W. Baird P. Davis R. H. Jr. Ferreri S. Knudtson M. Koraym A.. (2009). Health Benefits of Dietary Fiber. Nutr. Rev. 67 (4), 188–205. 10.1111/j.1753-4887.2009.00189.x 19335713

[B3] Aung T. Halsey J. Kromhout D. Gerstein H. C. Marchioli R. Tavazzi L.. (2018). Associations of Omega-3 Fatty Acid Supplement Use With Cardiovascular Disease Risks: Meta-Analysis of 10 Trials Involving 77 917 Individuals. JAMA Cardiol. 3 (3), 225–234. 10.1001/jamacardio.2017.5205 29387889 PMC5885893

[B4] Benakis C. Brea D. Caballero S. Faraco G. Moore J. Murphy M.. (2016). Commensal Microbiota Affects Ischemic Stroke Outcome by Regulating Intestinal γδ T Cells. Nat. Med. 22 (5), 516–523. 10.1038/nm.4068 27019327 PMC4860105

[B5] Blasco M. P. Chauhan A. Honarpisheh P. Ahnstedt H. d’Aigle J. Ganesan A.. (2020). Age-Dependent Involvement of Gut Mast Cells and Histamine in Post-Stroke Inflammation. J. Neuroinflamm. 17 (1), 160. 10.1186/s12974-020-01833-1 PMC7236952 32429999

[B6] Bothe M. K. Maathuis A. J. H. Bellmann S. van der Vossen J. Berressem D. Koehler A.. (2017). Dose-Dependent Prebiotic Effect of Lactulose in a Computer-Controlled *In Vitro* Model of the Human Large Intestine. Nutrients 9 (7), 767. 10.3390/nu9070767 PMC5537881 28718839

[B7] Brichacek A. L. Nwafor D. C. Benkovic S. A. Chakraborty S. Kenney S. M. Mace M. E.. (2020). Experimental Stroke Induces Chronic Gut Dysbiosis and Neuroinflammation in Male Mice. bioRxiv [Preprint] 2020.2004.2029.069575. 10.1101/2020.04.29.069575

[B8] Caso J. R. Pradillo J. M. Hurtado O. Lorenzo P. Moro M. A. Lizasoain I. (2007). Toll-Like Receptor 4 Is Involved in Brain Damage and Inflammation After Experimental Stroke. Circulation 115 (12), 1599–1608. 10.1161/circulationaha.106.603431 17372179

[B9] Cekanaviciute E. Fathali N. Doyle K. P. Williams A. M. Han J. Buckwalter M. S. (2014). Astrocytic Transforming Growth Factor-Beta Signaling Reduces Subacute Neuroinflammation After Stroke in Mice. Glia 62 (8), 1227–1240. 10.1002/glia.22675 24733756 PMC4061255

[B10] Chen R. Xu Y. Wu P. Zhou H. Lasanajak Y. Fang Y.. (2019). Transplantation of Fecal Microbiota Rich in Short Chain Fatty Acids and Butyric Acid Treat Cerebral Ischemic Stroke by Regulating Gut Microbiota. Pharmacol. Res. 148:104403. 10.1016/j.phrs.2019.104403 31425750

[B11] Chen X. Zhang Z. Hu Y. Cui J. Zhi X. Li X.. (2020). Lactulose Suppresses Osteoclastogenesis and Ameliorates Estrogen Deficiency-Induced Bone Loss in Mice. Aging Dis. 11 (3), 629–641. 10.14336/ad.2019.0613 32489707 PMC7220299

[B12] Cryan J. F. O’Riordan K. J. Cowan C. S. M. Sandhu K. V. Bastiaanssen T. F. S. Boehme M.. (2019). The Microbiota-Gut-Brain Axis. Physiol. Rev. 99 (4), 1877–2013. 10.1152/physrev.00018.2018 31460832

[B13] da Silva D. M. Martins J. L. R. de Oliveira D. R. Florentino I. F. da Silva D. P. B. Dos Santos F. C. A.. (2018). Effect of Allantoin on Experimentally Induced Gastric Ulcers: Pathways of Gastroprotection. Eur. J. Pharmacol. 821, 68–78. 10.1016/j.ejphar.2017.12.052 29277718

[B14] Durgan D. J. Lee J. McCullough L. D. Bryan R. M. Jr. (2019). Examining the Role of the Microbiota-Gut-Brain Axis in Stroke. Stroke 50 (8), 2270–2277. 10.1161/strokeaha.119.025140 31272315 PMC6646086

[B15] Eslami-Farsani M. Moslehi A. Hatami-Shahmir A. (2018). Allantoin Improves Histopathological Evaluations in a Rat Model of Gastritis. Physiol. Int. 105 (4), 325–334. 10.1556/2060.105.2018.4.30 30582339

[B16] Gao H. Liu S. (2017). Role of Uremic Toxin Indoxyl Sulfate in the Progression of Cardiovascular Disease. Life Sci. 185, 23–29. 10.1016/j.lfs.2017.07.027 28754616

[B17] Gao X. Cao Q. Cheng Y. Zhao D. Wang Z. Yang H.. (2018). Chronic Stress Promotes Colitis by Disturbing the Gut Microbiota and Triggering Immune System Response. Proc. Natl. Acad. Sci. U.S.A. 115 (13), E2960–e2969. 10.1073/pnas.1720696115 29531080 PMC5879702

[B18] Guan W. Zhao Y. Xu C. (2011). A Combined Treatment With Taurine and Intra-Arterial Thrombolysis in an Embolic Model of Stroke in Rats: Increased Neuroprotective Efficacy and Extended Therapeutic Time Window. Transl. Stroke Res. 2 (1), 80–91. 10.1007/s12975-010-0050-4 24323587

[B19] Hou L. Che Y. Sun F. Wang Q. (2018). Taurine Protects Noradrenergic Locus Coeruleus Neurons in a Mouse Parkinson’s Disease Model by Inhibiting Microglial M1 Polarization. Amino Acids 50 (5), 547–556. 10.1007/s00726-018-2547-1 29508060

[B20] Huang Y. Zhou J. Wang S. Xiong J. Chen Y. Liu Y.. (2020). Indoxyl Sulfate Induces Intestinal Barrier Injury Through IRF1-DRP1 Axis-Mediated Mitophagy Impairment. Theranostics 10 (16), 7384–7400. 10.7150/thno.45455 32641998 PMC7330852

[B21] Hung S. C. Kuo K. L. Wu C. C. Tarng D. C. (2017). Indoxyl Sulfate: A Novel Cardiovascular Risk Factor in Chronic Kidney Disease. J. Am. Heart Assoc. 6 (2), e005022. 10.1161/jaha.116.005022 28174171 PMC5523780

[B22] Innes J. K. Calder P. C. (2018). The Differential Effects of Eicosapentaenoic Acid and Docosahexaenoic Acid on Cardiometabolic Risk Factors: A Systematic Review. Int. J. Mol. Sci. 19 (2), 535. 10.3390/ijms19020532 PMC5855754 29425187

[B23] Jakaria M. Azam S. Haque M. E. Jo S. H. Uddin M. S. Kim I. S.. (2019). Taurine and Its Analogs in Neurological Disorders: Focus on Therapeutic Potential and Molecular Mechanisms. Redox Biol. 24, 101223. 10.1016/j.redox.2019.101223 31141786 PMC6536745

[B24] Jin R. Xiao A. Y. Liu S. Wang M. Li G. (2018). Taurine Reduces tPA (Tissue-Type Plasminogen Activator)-Induced Hemorrhage and Microvascular Thrombosis After Embolic Stroke in Rat. Stroke 49 (7), 1708–1718. 10.1161/strokeaha.118.020747 29844028 PMC6019579

[B25] Kand’ár R. Záková P. Muzáková V. (2006). Monitoring of Antioxidant Properties of Uric Acid in Humans for a Consideration Measuring of Levels of Allantoin in Plasma by Liquid Chromatography. Clin. Chim. Acta 365 (1-2), 249–256. 10.1016/j.cca.2005.09.002 16194528

[B26] Katakawa M. Fukuda N. Tsunemi A. Mori M. Maruyama T. Matsumoto T.. (2016). Taurine and Magnesium Supplementation Enhances the Function of Endothelial Progenitor Cells Through Antioxidation in Healthy Men and Spontaneously Hypertensive Rats. Hypertens. Res. 39 (12), 848–856. 10.1038/hr.2016.86 27412799

[B27] Lambertsen K. L. Finsen B. Clausen B. H. (2019). Post-Stroke Inflammation-Target or Tool for Therapy? Acta Neuropathol. 137 (5), 693–714. 10.1007/s00401-018-1930-z 30483945 PMC6482288

[B28] Li N. Wang X. Sun C. Wu X. Lu M. Si Y.. (2019). Change of Intestinal Microbiota in Cerebral Ischemic Stroke Patients. BMC Microbiol. 19 (1), 191. 10.1186/s12866-019-1552-1 31426765 PMC6700817

[B29] Liu L. Locascio L. M. Doré S. (2019). Critical Role of Nrf2 in Experimental Ischemic Stroke. Front. Pharmacol. 10, 153. 10.3389/fphar.2019.00153 30890934 PMC6411824

[B30] Llull L. Laredo C. Renú A. Pérez B. Vila E. Obach V.. (2015). Uric Acid Therapy Improves Clinical Outcome in Women With Acute Ischemic Stroke. Stroke 46 (8), 2162–2167. 10.1161/strokeaha.115.009960 26159792

[B31] Maiuolo J. Oppedisano F. Gratteri S. Muscoli C. Mollace V. (2016). Regulation of Uric Acid Metabolism and Excretion. Int. J. Cardiol. 213, 8–14. 10.1016/j.ijcard.2015.08.109 26316329

[B32] Ma D. Wang A. C. Parikh I. Green S. J. Hoffman J. D. Chlipala G.. (2018). Ketogenic Diet Enhances Neurovascular Function With Altered Gut Microbiome in Young Healthy Mice. Sci. Rep. 8 (1), 6670. 10.1038/s41598-018-25190-5 29703936 PMC5923270

[B33] Mao B. Li D. Ai C. Zhao J. Zhang H. Chen W. (2016). Lactulose Differently Modulates the Composition of Luminal and Mucosal Microbiota in C57BL/6J Mice. J. Agric. Food Chem. 64 (31), 6240–6247. 10.1021/acs.jafc.6b02305 27438677

[B34] Nakase T. Sasaki M. Suzuki A. (2015). Eicosapentaenoic Acid as Long-Term Secondary Prevention After Ischemic Stroke. Clin. Transl. Med. 4 (1), 62. 10.1186/s40169-015-0062-5 26084813 PMC4471066

[B35] Nooshkam M. Babazadeh A. Jooyandeh H. (2018). Lactulose: Properties, Techno-Functional Food Applications, and Food Grade Delivery System. Trends Food Sci. Technol. 80, 23–34. 10.1016/j.tifs.2018.07.028

[B36] Obrosova I. G. Fathallah L. Stevens M. J. (2001). Taurine Counteracts Oxidative Stress and Nerve Growth Factor Deficit in Early Experimental Diabetic Neuropathy. Exp. Neurol. 172 (1), 211–219. 10.1006/exnr.2001.7789 11681853

[B37] Ohsawa Y. Hagiwara H. Nishimatsu S. I. Hirakawa A. Kamimura N. Ohtsubo H.. (2019). Taurine Supplementation for Prevention of Stroke-Like Episodes in MELAS: A Multicentre, Open-Label, 52-Week Phase III Trial. J. Neurol. Neurosurg. Psychiatry 90 (5), 529–536. 10.1136/jnnp-2018-317964 29666206 PMC6581075

[B38] Schaffer S. Kim H. W. (2018). Effects and Mechanisms of Taurine as a Therapeutic Agent. Biomol. Ther. (Seoul) 26 (3), 225–241. 10.4062/biomolther.2017.251 29631391 PMC5933890

[B39] Schumann C. (2002). Medical, Nutritional and Technological Properties of Lactulose. An Update. Eur. J. Nutr. 41 Suppl 1, I17–I25. 10.1007/s00394-002-1103-6 12420112

[B40] Seet R. C. Lee C. Y. Chan B. P. Sharma V. K. Teoh H. L. Venketasubramanian N.. (2011). Oxidative Damage in Ischemic Stroke Revealed Using Multiple Biomarkers. Stroke 42 (8), 2326–2329. 10.1161/strokeaha.111.618835 21700941

[B41] Shi K. Tian D. C. Li Z. G. Ducruet A. F. Lawton M. T. Shi F. D. (2019). Global Brain Inflammation in Stroke. Lancet Neurol. 18 (11), 1058–1066. 10.1016/s1474-4422(19)30078-x 31296369

[B42] Singh V. Roth S. Llovera G. Sadler R. Garzetti D. Stecher B.. (2016). Microbiota Dysbiosis Controls the Neuroinflammatory Response After Stroke. J. Neurosci. 36 (28), 7428–7440. 10.1523/jneurosci.1114-16.2016 27413153 PMC6705544

[B43] Spychala M. S. Venna V. R. Jandzinski M. Doran S. J. Durgan D. J. Ganesh B. P.. (2018). Age-Related Changes in the Gut Microbiota Influence Systemic Inflammation and Stroke Outcome. Ann. Neurol. 84 (1), 23–36. 10.1002/ana.25250 29733457 PMC6119509

[B44] Stanley D. Mason L. J. Mackin K. E. Srikhanta Y. N. Lyras D. Prakash M. D.. (2016). Translocation and Dissemination of Commensal Bacteria in Post-Stroke Infection. Nat. Med. 22 (11), 1277–1284. 10.1038/nm.4194 27694934

[B45] Sun M. Zhao Y. M. Gu Y. Xu C. (2012). Therapeutic Window of Taurine Against Experimental Stroke in Rats. Transl. Res. 160 (3), 223–229. 10.1016/j.trsl.2012.02.007 22683413

[B46] Tan C. Wu Q. Wang H. Gao X. Xu R. Cui Z.. (2020). Dysbiosis of Gut Microbiota and Short-Chain Fatty Acids in Acute Ischemic Stroke and the Subsequent Risk for Poor Functional Outcomes. JPEN J. Parenter. Enteral Nutr. 45 (3), 518–529. 10.1002/jpen.1861 32473086 PMC8048557

[B47] Vanholder R. Schepers E. Pletinck A. Nagler E. V. Glorieux G. (2014). The Uremic Toxicity of Indoxyl Sulfate and P-Cresyl Sulfate: A Systematic Review. J. Am. Soc. Nephrol. 25 (9), 1897–1907. 10.1681/asn.2013101062 24812165 PMC4147984

[B48] Waisman A. Hauptmann J. Regen T. (2015). The Role of IL-17 in CNS Diseases. Acta Neuropathol. 129 (5), 625–637. 10.1007/s00401-015-1402-7 25716179

[B49] Wang H. X. Wang Y. P. (2016). Gut Microbiota-Brain Axis. Chin. Med. J. (Engl.) 129 (19), 2373–2380. 10.4103/0366-6999.190667 27647198 PMC5040025

[B50] Winek K. Engel O. Koduah P. Heimesaat M. M. Fischer A. Bereswill S.. (2016). Depletion of Cultivatable Gut Microbiota by Broad-Spectrum Antibiotic Pretreatment Worsens Outcome After Murine Stroke. Stroke 47 (5), 1354–1363. 10.1161/strokeaha.115.011800 27056982 PMC4839545

[B51] Yan T. Chen Z. Chopp M. Venkat P. Zacharek A. Li W.. (2020). Inflammatory Responses Mediate Brain-Heart Interaction After Ischemic Stroke in Adult Mice. J. Cereb. Blood Flow Metab. 40 (6), 1213–1229. 10.1177/0271678x18813317 30465612 PMC7238382

[B52] Zhai S. Zhu L. Qin S. Li L. (2018). Effect of Lactulose Intervention on Gut Microbiota and Short Chain Fatty Acid Composition of C57BL/6J Mice. Microbiologyopen 7 (6), e00612. 10.1002/mbo3.612 29575825 PMC6291785

[B53] Zhai X. Chen X. Shi J. Shi D. Ye Z. Liu W.. (2013). Lactulose Ameliorates Cerebral Ischemia-Reperfusion Injury in Rats by Inducing Hydrogen by Activating Nrf2 Expression. Free Radic. Biol. Med. 65, 731–741. 10.1016/j.freeradbiomed.2013.08.004 23954468

[B54] Zhang D. Chia C. Jiao X. Jin W. Kasagi S. Wu R.. (2017). D-Mannose Induces Regulatory T Cells and Suppresses Immunopathology. Nat. Med. 23 (9), 1036–1045. 10.1038/nm.4375 28759052 PMC12180587

[B55] Zhang Z. Chen X. Zhao J. Tian C. Wei X. Li H.. (2019a). Effects of a Lactulose-Rich Diet on Fecal Microbiome and Metabolome in Pregnant Mice. J. Agric. Food Chem. 67 (27), 7674–7683. 10.1021/acs.jafc.9b01479 31132256

[B56] Zhang Z. Zhao J. Tian C. Chen X. Li H. Wei X.. (2019b). Targeting the Gut Microbiota to Investigate the Mechanism of Lactulose in Negating the Effects of a High-Salt Diet on Hypertension. Mol. Nutr. Food Res. 63 (11), e1800941. 10.1002/mnfr.201800941 30825362

[B57] Zhao Q. Yan T. Chopp M. Venkat P. Chen J. (2020). Brain-Kidney Interaction: Renal Dysfunction Following Ischemic Stroke. J. Cereb. Blood Flow Metab. 40 (2), 246–262. 10.1177/0271678x19890931 31766979 PMC7370616

