# Supplementary material for: Lactulose Improves Neurological Outcomes by Repressing Harmful Bacteria and Regulating Inflammatory Reactions in Mice After Stroke
Source: Front Cell Infect Microbiol. 2021 Jul 13;11:644448. doi: 10.3389/fcimb.2021.644448 (PMC8313872; doi:10.3389/fcimb.2021.644448)

**Supplemental Figure 1**. gate strategy


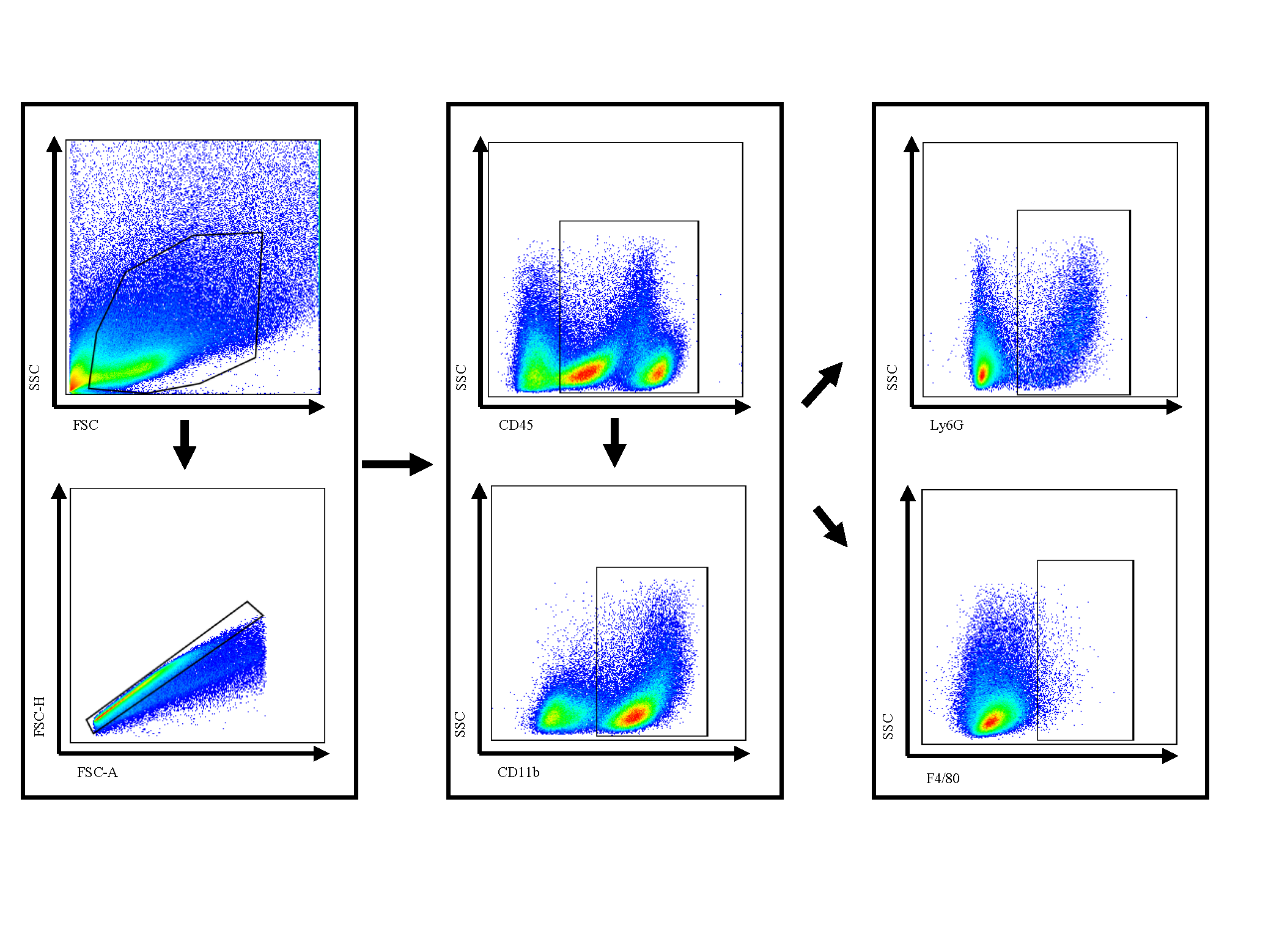


**Supplemental Figure 2**:SCFA level among three groups


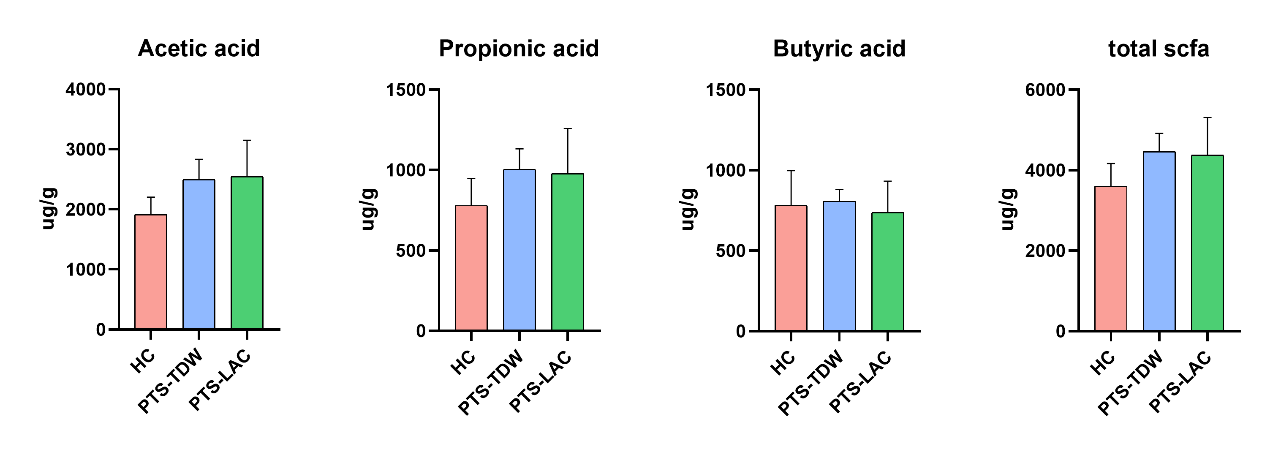


- We also examined SCFA alteration in the feces. Consistent with a previous study(Zhai et al., 2018),no obvious differences in SCFA concentrations were observed among the three groups after lactulose supplementation (Supplemental Figure 2 ), although other studies found that lactulose can increase particular species of SCFAs, such as acetate, butyrate(Bothe et al., 2017), and even total SCFAs(Zheng et al., 2018)

**Supplemental Figure 3**: Four expression patterns (profile 0–3) of hierarchical clustering in the positive and negative modes.

Pos:


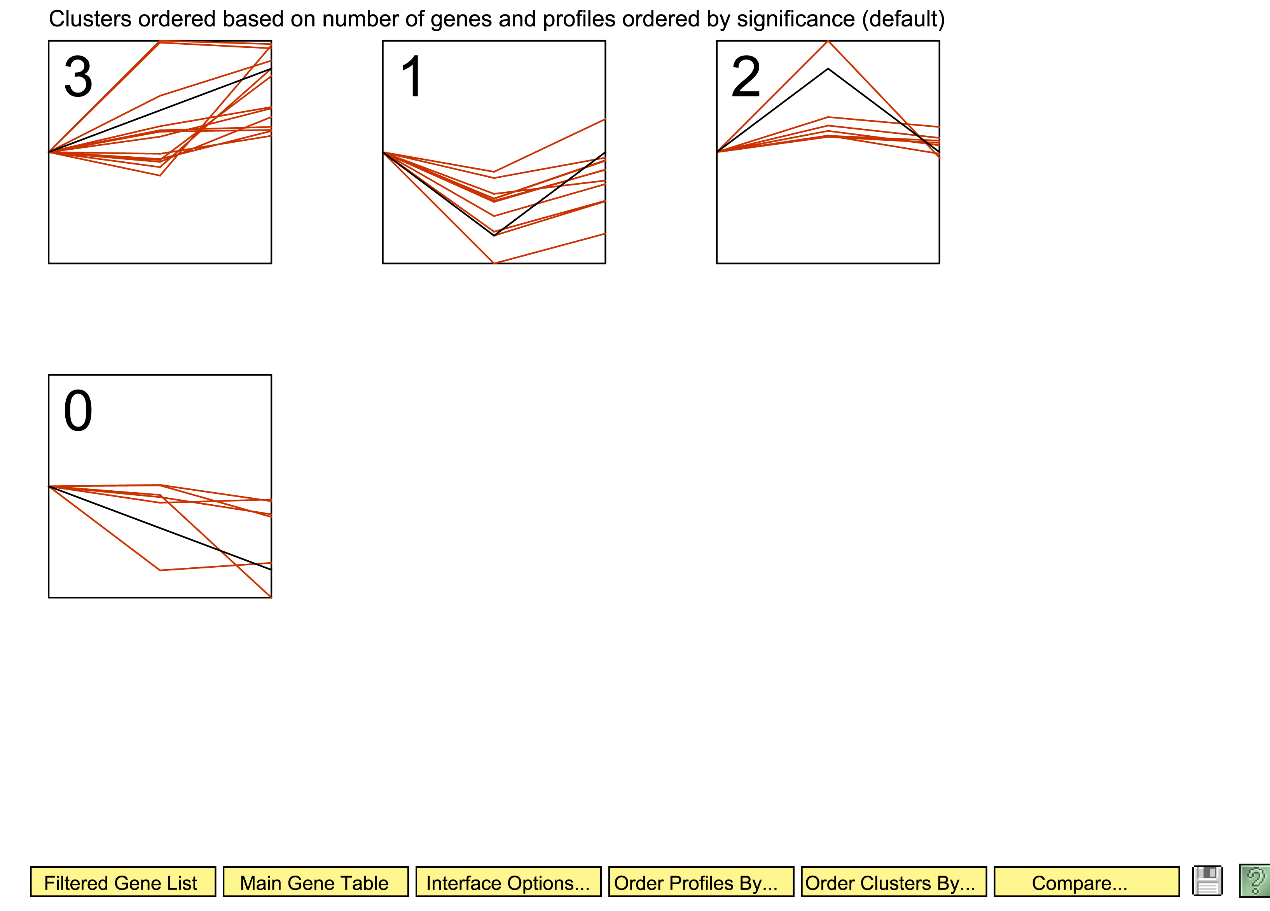


Neg:


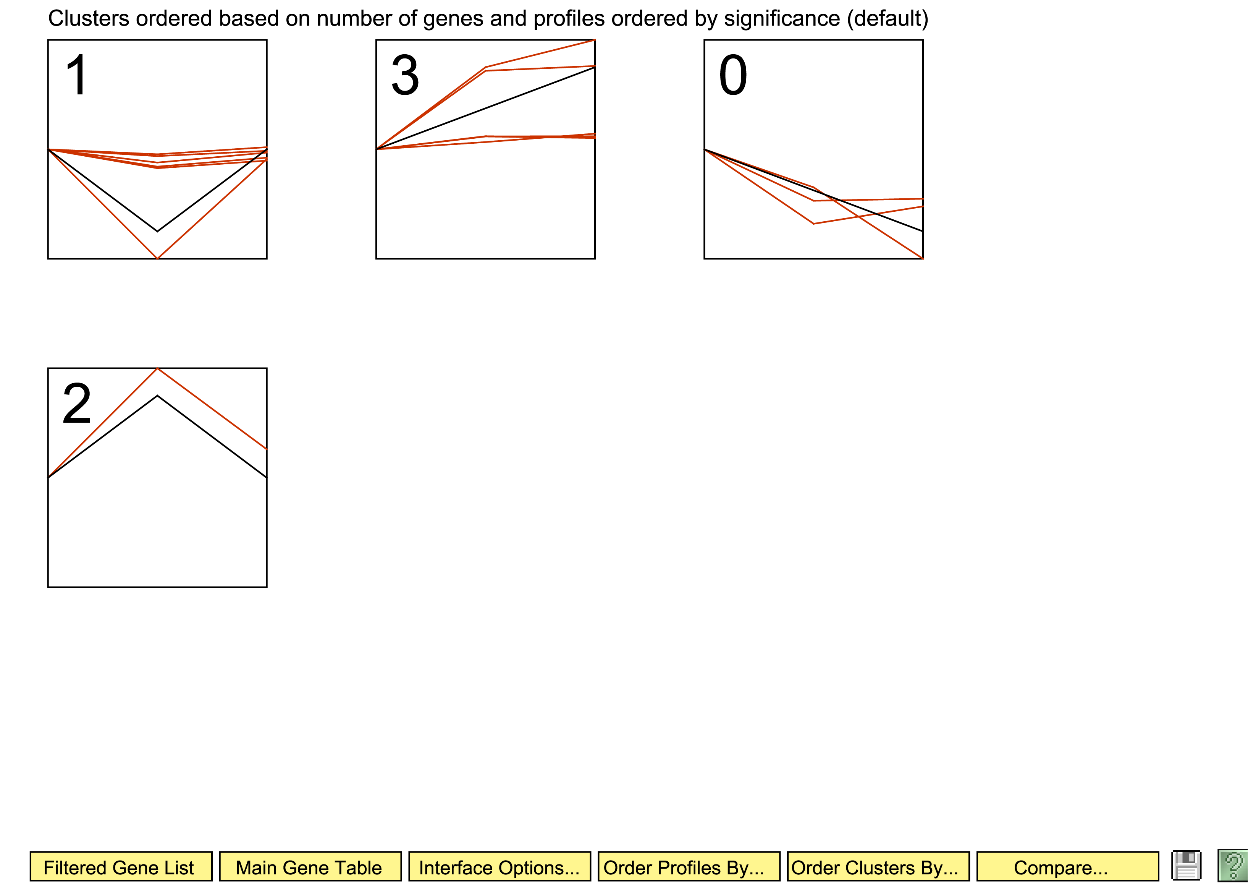


**Supplemental Figure 4**: The heatmap of clustering for genus abundance


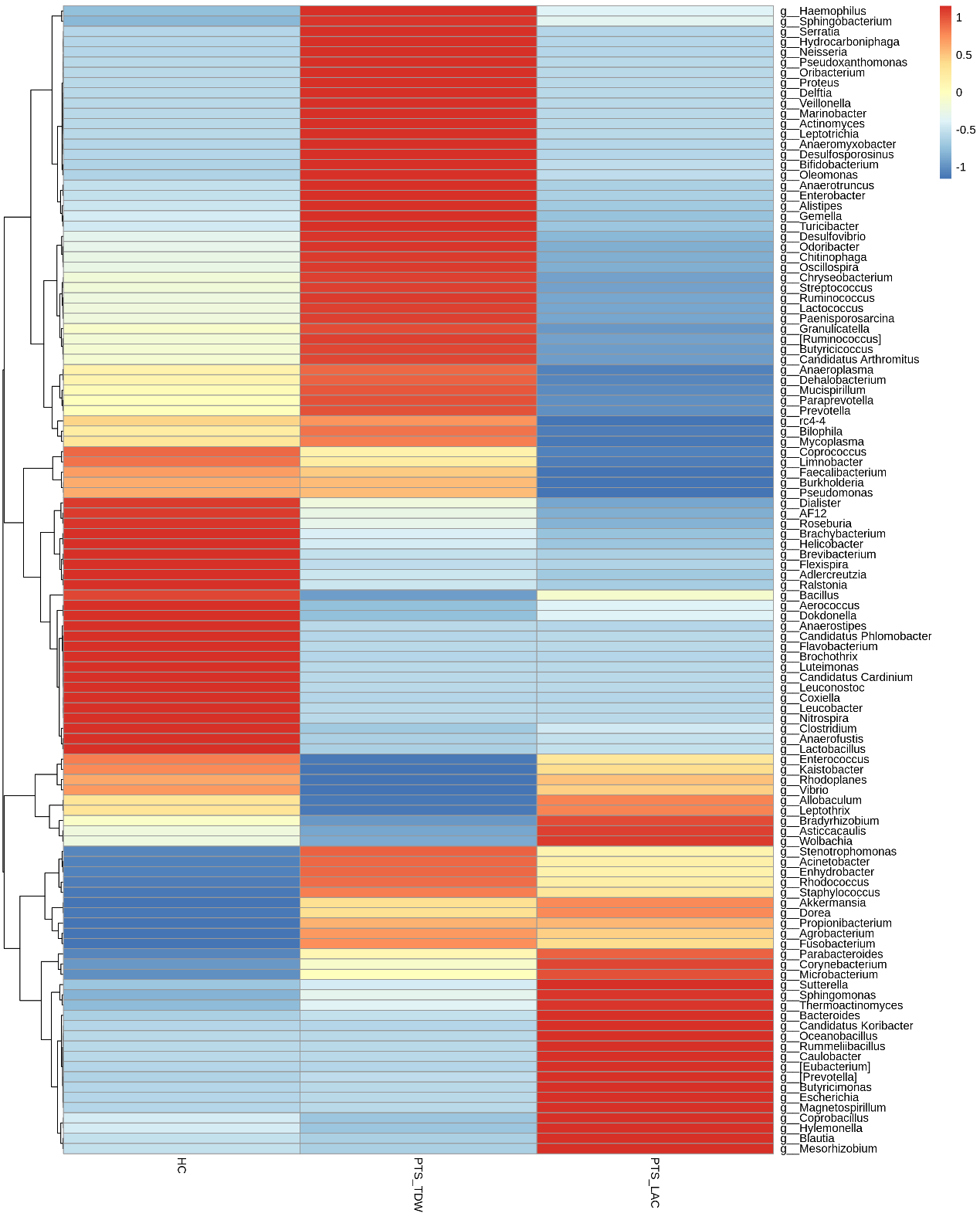


**Supplemental Figure 5**: The heatmap of clustering for phylum abundance


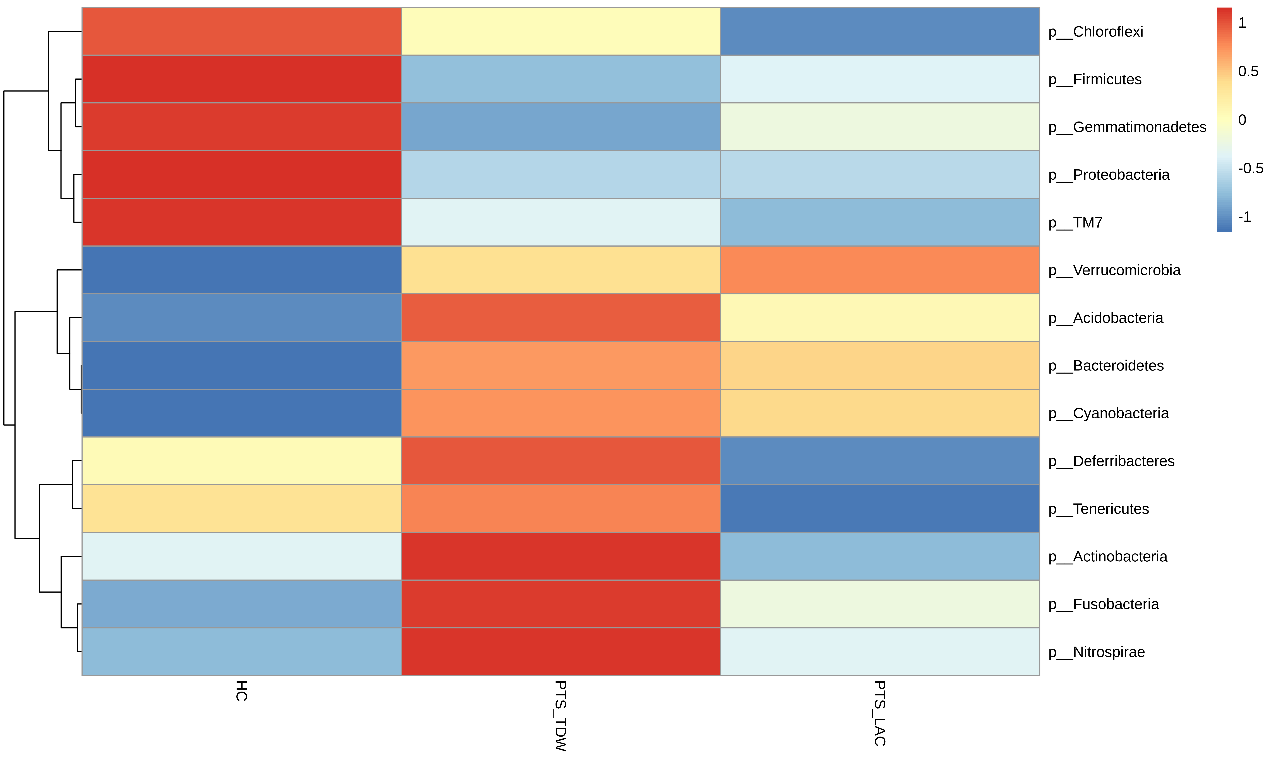

Supplement: Supplementary file 1 [file DataSheet_1.zip › supplemental figure &legend.docx]
