# Supplementary figures and images for: Lactulose Improves Neurological Outcomes by Repressing Harmful Bacteria and Regulating Inflammatory Reactions in Mice After Stroke
Source: Front Cell Infect Microbiol. 2021 Jul 13;11:644448. doi: 10.3389/fcimb.2021.644448 (PMC8313872; doi:10.3389/fcimb.2021.644448)

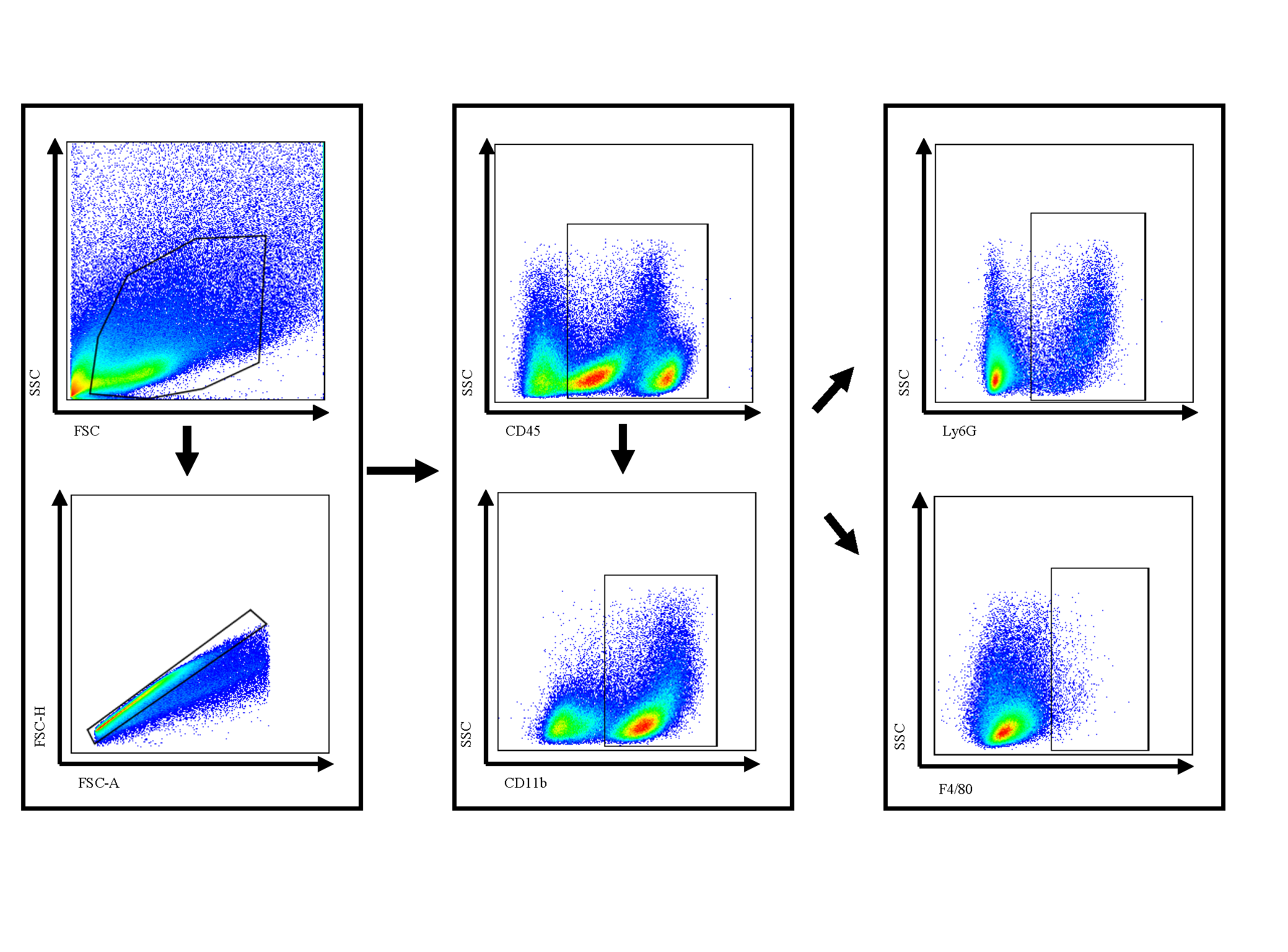

Supplement: Supplementary file 1 [file DataSheet_1.zip › FC gate strategy.tiff]

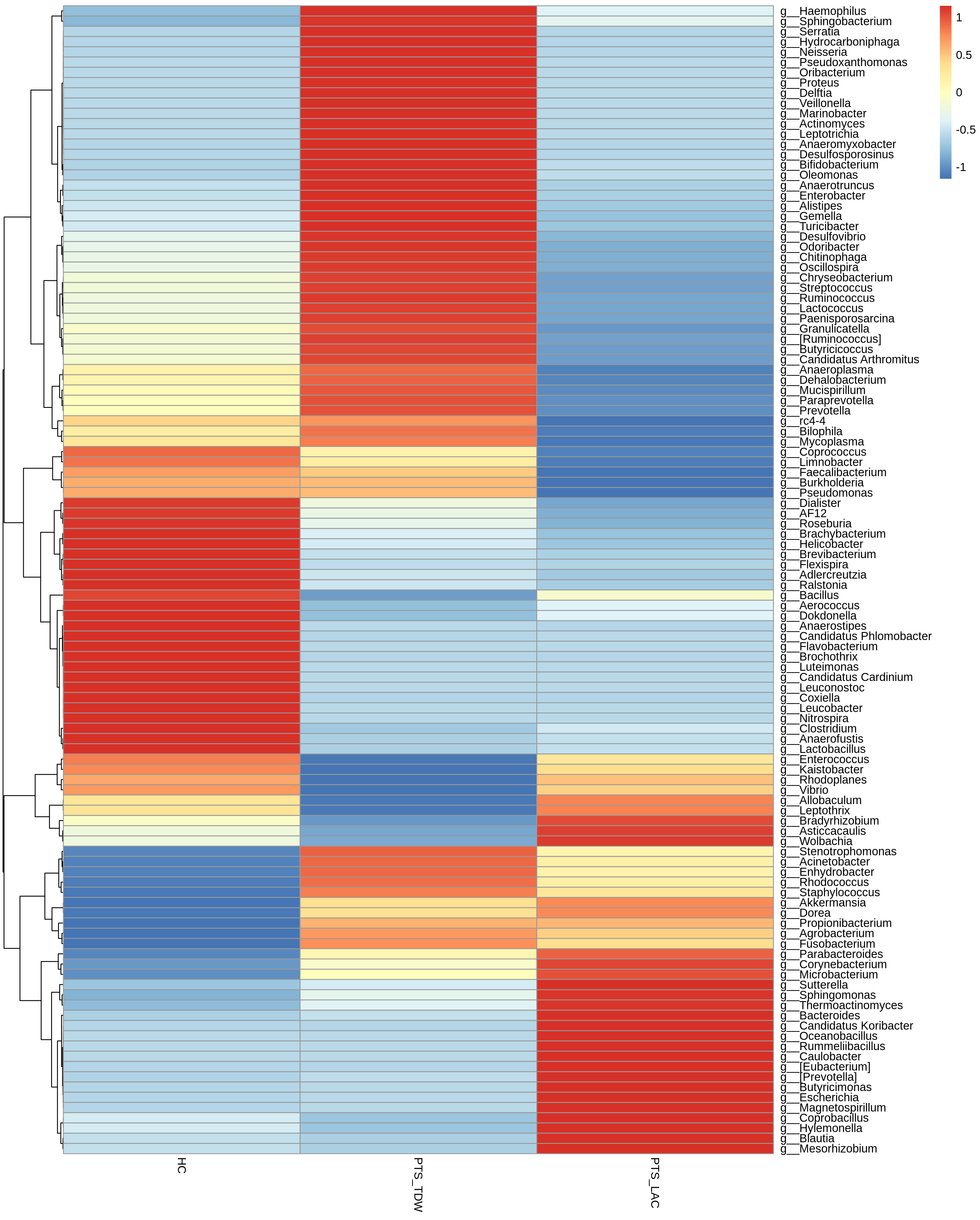

Supplement: Supplementary file 1 [file DataSheet_1.zip › heatmap of clustering for genus abundance.png]

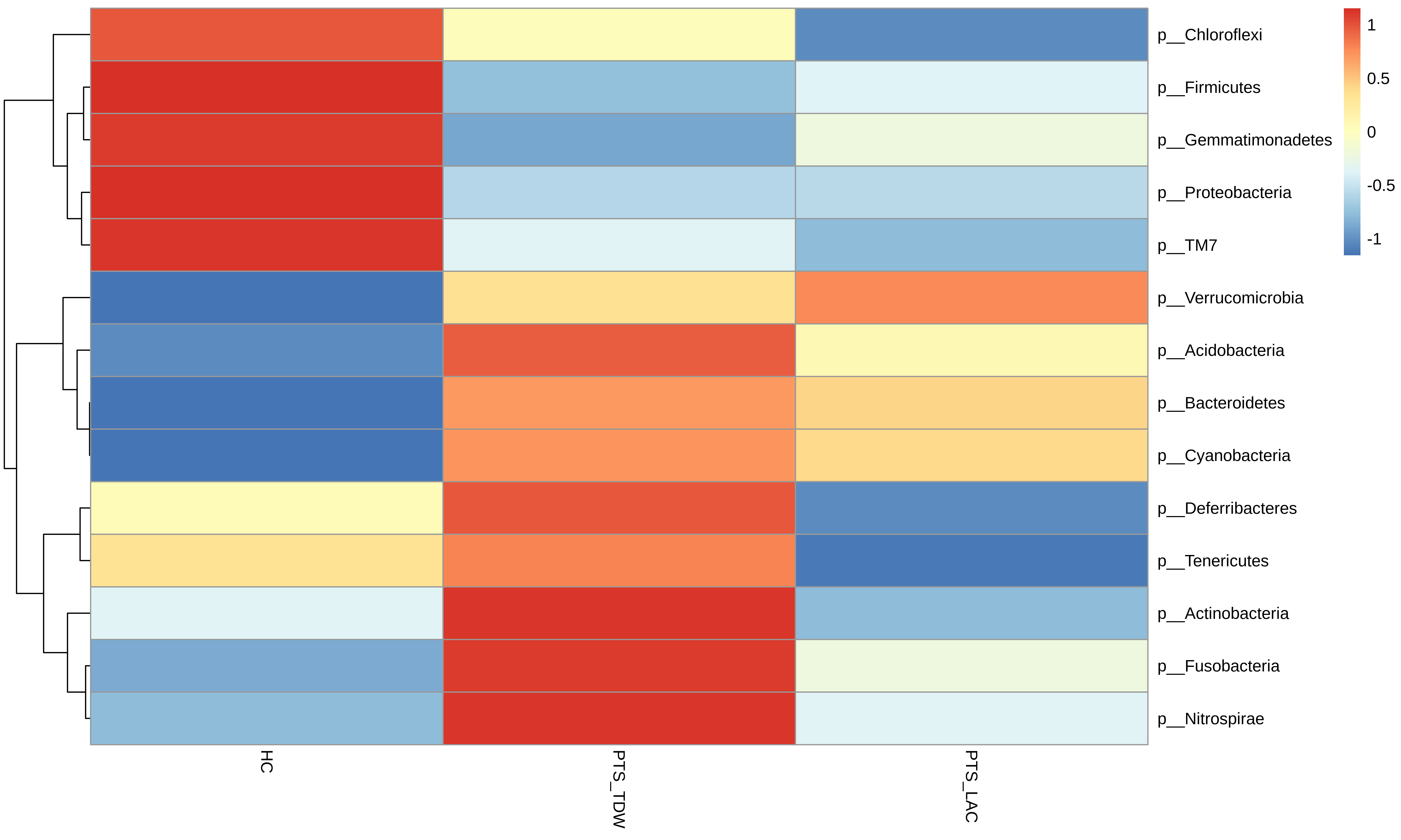

Supplement: Supplementary file 1 [file DataSheet_1.zip › heatmap of clustering for phylum abundance.png]

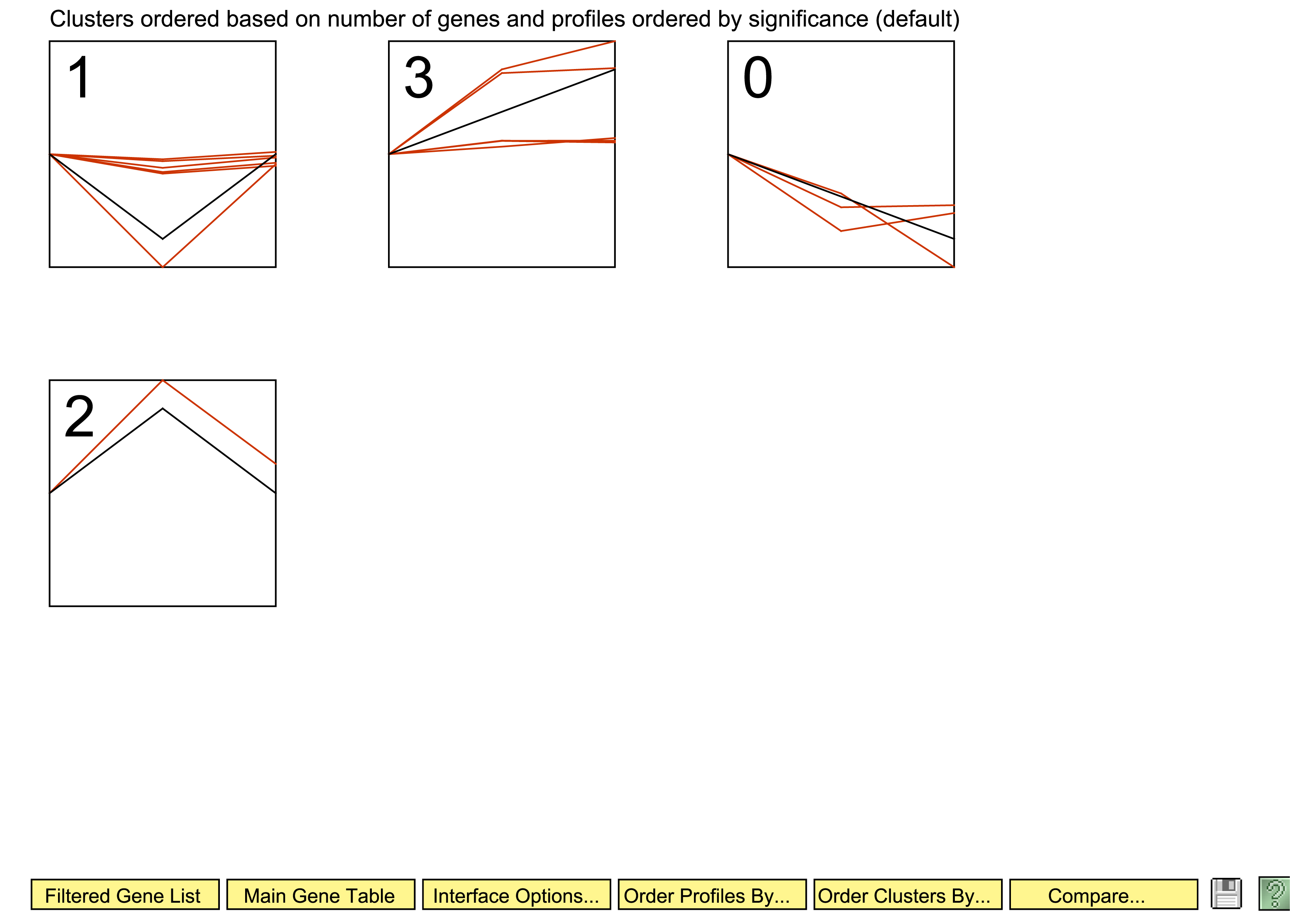

Supplement: Supplementary file 1 [file DataSheet_1.zip › neg-cluster.tif]

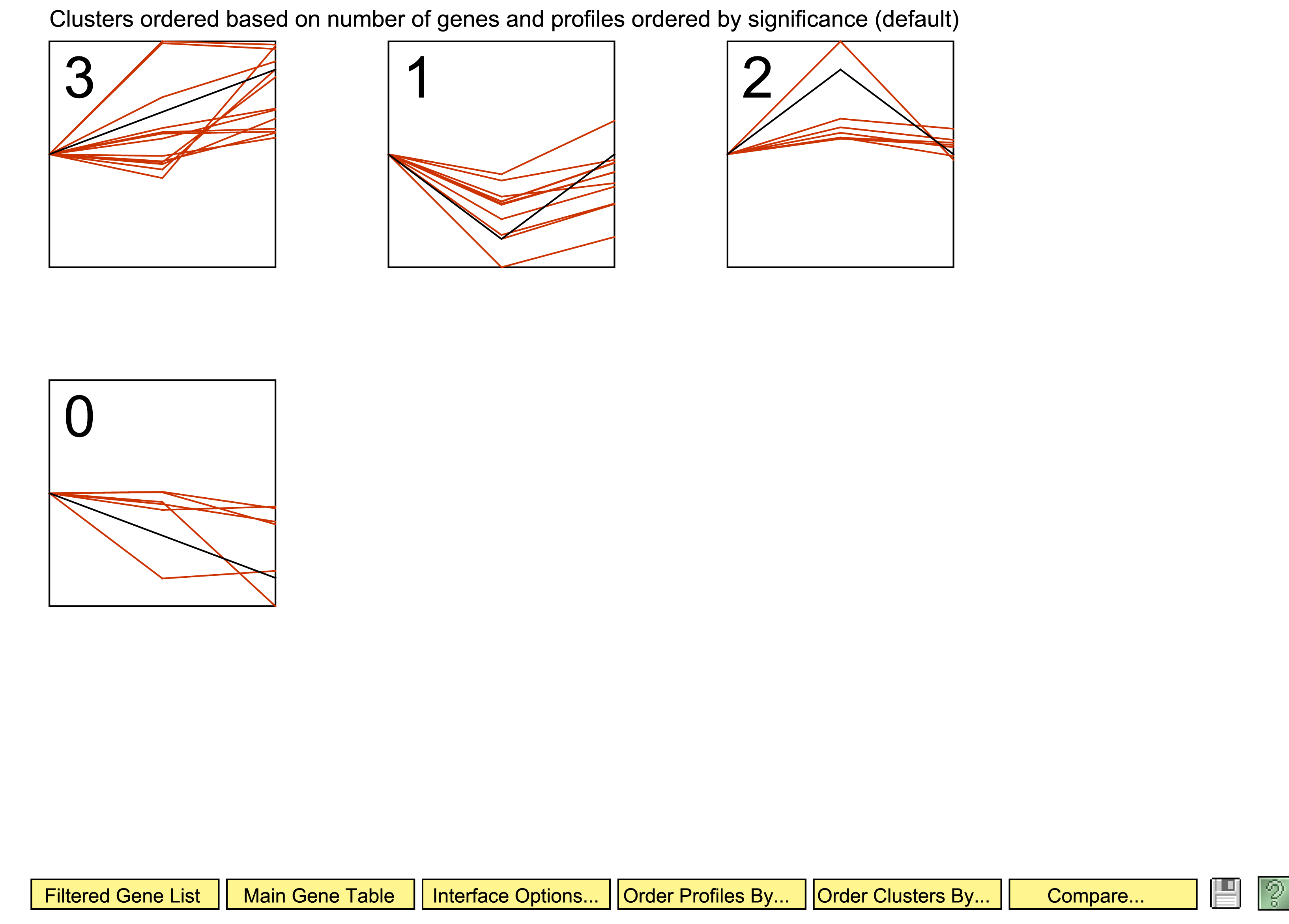

Supplement: Supplementary file 1 [file DataSheet_1.zip › pos-cluster.tif]

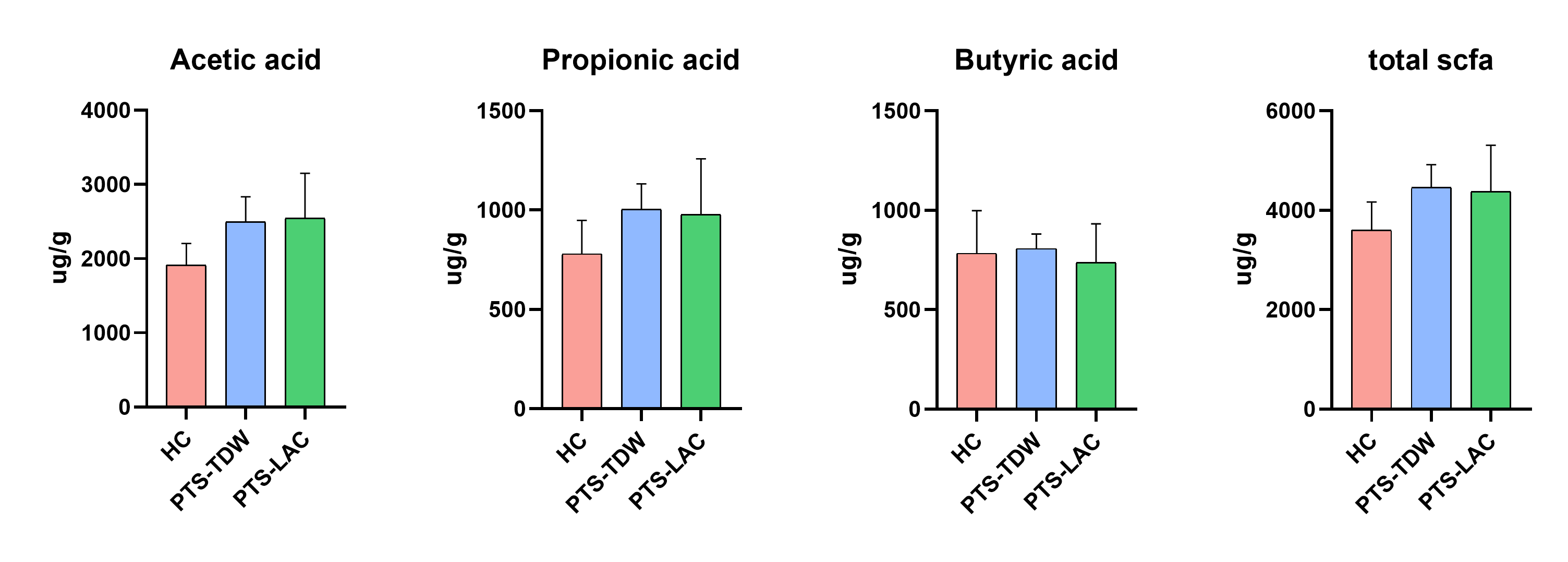

Supplement: Supplementary file 1 [file DataSheet_1.zip › SCFA.tif]
